# *Fusobacterium nucleatum* in Health and Disease

**DOI:** 10.1002/mco2.70465

**Published:** 2025-11-09

**Authors:** Xinyi Yang, Shutian Zhang, Tingting Ning, Jing Wu

**Affiliations:** ^1^ Department of Gastroenterology, Beijing Friendship Hospital, National Clinical Research Center For Digestive Disease, Beijing Digestive Disease Center, State Key Laboratory of Digestive Health Capital Medical University Beijing China

**Keywords:** *Fusobacterium nucleatum*, health, inflammation, malignancy, microbiota

## Abstract

*Fusobacterium nucleatum* (*F. nucleatum*), a prevalent anaerobe primarily colonizing the oral cavity, functions as both a commensal biofilm architect and an opportunistic pathogen. Increasing evidence implicates it in a wide spectrum of inflammatory diseases and malignancies. This review synthesizes current knowledge with emphasis on subspecies‐specific functional distinctions and pathological relevance. Beyond its established roles in oral inflammation, atherosclerosis, and adverse pregnancy outcomes, *F. nucleatum* is emerging as a key oncomicrobe, particularly in colorectal cancer. It can translocate from the oral niche to extra‐oral sites, where it fuels chronic inflammation and promotes tumor initiation, progression, metastasis, and therapy resistance. Pathogenic mechanisms include host transmission pathways, adhesion and colonization strategies, induction of inflammatory cascades, enhancement of cellular proliferation and metastatic potential, immune modulation, and contribution to therapeutic resistance. We further explore its dynamic interactions with host cells and microbial communities, highlighting how microbial synergy and antagonism shape disease outcomes. Current and emerging therapeutic and preventive strategies targeting *F. nucleatum* are systematically evaluated. A nuanced understanding of the context‐dependent pathogenicity of *F. nucleatum* and its ecological interactions is critical for advancing the development of robust diagnostic biomarkers and precision therapeutics aimed at mitigating its disease burden.

## Graphical abstract

*Fusobacterium nucleatum* (*Fn*), an oral commensal and opportunistic pathogen, primarily colonizes the oral cavity. It contributes to multiple nonmalignant diseases and cancers—especially colorectal cancer—through interaction with the host and other microbes, leading to inflammation, enhanced cellular proliferation, metastatic progression, and immune evasion. Emerging interventions aim to eliminate *Fn*, mitigate its pathogenic effects, and restore host–microbiome balance. Created with BioRender.com.

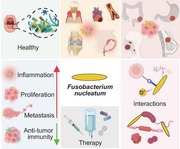

## 1 Introduction

*Fusobacterium nucleatum* (*F. nucleatum*), a Gram‐negative, nonmotile, anaerobic bacterium, was first described by Knorr in 1922 [1]. As the type species of the *Fusobacterium* genus, *F. nucleatum* is a key oral commensal, playing an essential role in dental biofilm formation and gingival plaque development through coaggregation with diverse oral microorganisms [2]. However, its pathogenic potential has recently attracted significant scientific attention, particularly due to its involvement in various oral and extra‐oral diseases.

While *F. nucleatum* has long been recognized as an oral pathogen, implicated in conditions such as periodontal diseases, recent evidence has linked *F. nucleatum* to a range of extra‐oral infections, including atherosclerosis, inflammatory bowel disease (IBD), and adverse pregnancy outcomes (APOs). Notably, its association with several malignancies, especially colorectal cancer (CRC), has propelled it into the spotlight [3, 4, 5, 6]. In these malignancies, *F. nucleatum* is not only enriched but also contributes to tumor progression, with ongoing investigations focusing on its virulence factors and the metabolites that promote its pathogenicity.

Despite these advances, significant gaps remain in our understanding of *F. nucleatum*’s precise role in disease. Current research has highlighted the heterogeneity among *F. nucleatum* subspecies, with emerging evidence suggesting distinct ecological niches and suggesting the reclassification of certain subspecies as independent species based on genomic data [7]. A long‐standing question is whether *F. nucleatum* acts as a causal agent in carcinogenesis or merely as an opportunistic pathogen exploiting the tumor microenvironment (TME). This review aims to systematically elucidate the role of *F. nucleatum* and establish a roadmap for future investigations.

Here, we synthesize current knowledge on *F. nucleatum*’s role in both health and disease, focusing on its association with both malignant and nonmalignant diseases. We explore its pathogenic mechanisms, including colonization, inflammation induction, tumor promotion, metastasis facilitation, immune modulation, and therapy resistance. This review also highlights the distinct ecological specializations of *F. nucleatum* subspecies and their interactions with other microbiota. Furthermore, we evaluate existing therapeutic strategies targeting *F. nucleatum* and propose future directions for research to address unresolved questions.

## 2 Taxonomy, Phylogeny, and Ecological Distribution of *F. nucleatum*

**FIGURE 1:**
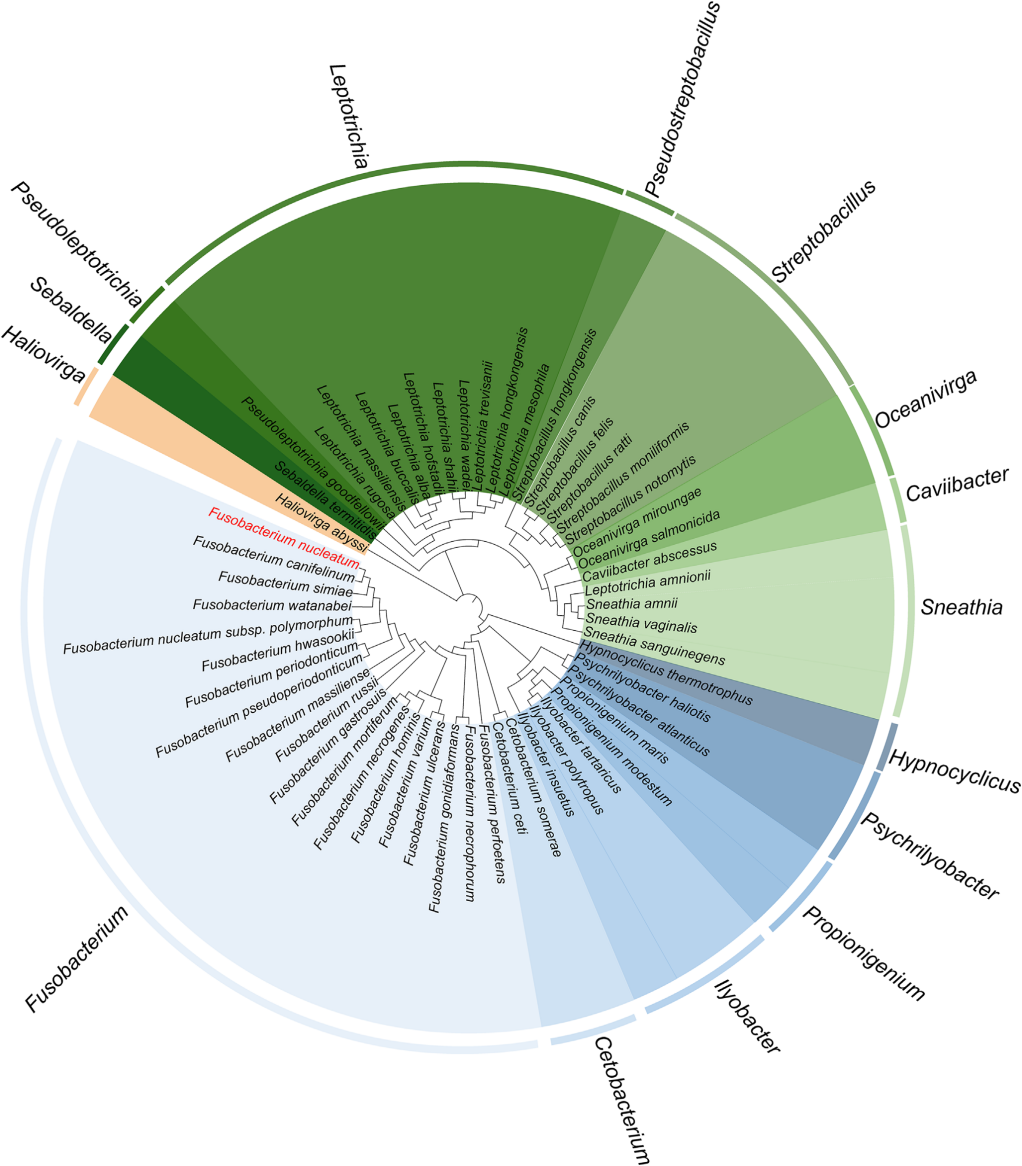
A phylogenetic tree of Fusobacteriales based upon the 16S rRNA gene sequences. The order Fusobacteriales comprises three families and 15 genera, with *Fusobacterium nucleatum* classified within the genus *Fusobacterium*. Distinct colors indicate different families, and color shades distinguish individual genera within each family. Constructed with MEGA 7 and visualized using the Interactive Tree of Life (iTOL) online tool.

*Fusobacterium* is one of 15 genera within the phylum Fusobacteriota (formerly known as Fusobacteria), which encompasses a single class (Fusobacteriia) and a single order (Fusobacteriales), further divided into three families: Fusobacteriaceae, Leptotrichiaceae, and Haliovirgaceae (Figure 1). Members of this genus are primarily Gram‐negative, anaerobic, nonspore‐forming, usually nonmotile, rod‐shaped bacteria, characterized by distinctive metabolic capabilities. These bacteria are typically associated with the mucosa of both humans and animals, commonly found in the human oral cavity, as well as in the human and animal gastrointestinal tract (GIT) and female urogenital tract [8, 9].

Members of *Fusobacterium* can be classified to three main clusters according to their specific conserved signature indels in proteins. *F. nucleatum* belongs to clade I for its slender and spindle‐shaped morphology with tapered or pointed ends. This species is notably involved in oral plaque formation and is associated with various diseases. Phylogenetic analysis has significantly enhanced our understanding of the genus *Fusobacterium* (Figure 1). Apart from *F. nucleatum*, more than 10 species within *Fusobacterium* are identified as pathogens affecting both humans and animals. Heterogeneity extensively exists in the genus *Fusobacterium* and subspecies of *F. nucleatum* [10]. It is noteworthy that *F. nucleatum* subspecies *animalis* (*Fna*), *F. nucleatum* subspecies *nucleatum* (*Fnn*), *F. nucleatum* subspecies *polymorphum* (*Fnp*), and *F. nucleatum* subspecies *vincentii* (*Fnv*) (inclusive of subspecies *fusiforme*) used to be classified as *F. nucleatum*, but reclassification into separate species has been proposed due to sufficient genetic divergence based on genome sequencing [11]. This reclassification remains a topic of debate, mainly because 16S rRNA sequence analysis has long been the primary method for defining bacterial species, traditionally requiring a 3% genetic divergence threshold to establish new species. However, the observed divergence among these four subspecies within their 16S rRNA genes is only 0.6–1.9%. Nevertheless, *Fna* and *Fnv* have been officially reclassified as *Fusobacterium animalis* sp. *Nov*. and *Fusobacterium vincentii* sp. *Nov*. per IJSEM (International Journal of Systematic and Evolutionary Microbiology) Validation List no 204 [12]. This review retains traditional nomenclature for practical utility, literature citation accuracy, and species/subspecies risk assessment. The classification of *F. nucleatum* remains dynamic, driven by the continuous emergence of novel genomic and phylogenetic evidence. Now more subspecies and distinct clades within *Fna* have been reported [13, 14]. Among these subspecies, *Fnn* strains are widely employed as model organisms in experiments, especially strains ATCC 25586, derived from a cervicofacial abscess, and ATCC 23726, cultured from urogenital tract specimens [12]. To provide a comprehensive overview of their pathogenic roles, a summary of their hosts and associated pathogenicity is presented in Table 1.

**TABLE 1:** A summary of the host and pathogenicity of *Fusobacterium* species and subspecies.

Species/subspecies	Host	Origin/diseases	References
*Fnn*	Human	Oral and extra‐oral diseases like appendicitis, IBD, CRC	[2]
*Fna*	Animal, human	Inflammatory periodontal diseases, APOs, CRC, abscess	[15]
*Fnv*	Human	Acute necrotizing ulcerative gingivitis, Vincent's angina	[16]
*Fnp*	Human	Supragingival biofilm, dental plaque, abscess	[17]
*Fusobacterium canifelinum*	Cats and dogs, human	Periodontitis	[18, 19]
*Fusobacterium simiae*	Macaques, human	Macaques dental plaque, human intra‐abdominal abscess	[20]
*Fusobacterium watanabei*	Human	Human pus, ascites fluid, pleural effusion	[21]
*Fusobacterium hwasookii*	Human	Periodontitis lesion	[22]
*Fusobacterium periodonticum*	Human	Periodontitis, oral and esophageal cancer, CRC, bronchiectasis, vertebral osteomyelitis	[23, 24, 25, 26, 27]
*Fusobacterium pseudoperiodonticum*	Human	Subgingival plaque of gingivitis lesion	[28]
*Fusobacterium massiliense*	Human	Liquid duodenum sample	[29]
*Fusobacterium russii*	Animal, human	Periodontal diseases, ulcer	[30]
*Fusobacterium gastrosuis*	Pigs	Gastritis, gastric ulcer disease	[31]
*Fusobacterium mortiferum*	Human	Septicemia, abscess	[32]
*Fusobacterium necrogenes*	Animals, human	Periodontal diseases	[9, 33]
*Fusobacterium hominis*	Human	Fresh fecal sample	[34]
*Fusobacterium varium*	Animal, human	IBD, Fournier's gangrene, abscess	[35, 36]
*Fusobacterium ulcerans*	Human	Tropical ulcers	[37]
*Fusobacterium gonidiaformans*	Human, mouse	Bacteremia, ulcerative colitis, CRC	[38, 39, 40]
*Fusobacterium necrophorum* subsp*. necrophorum*	Animal, human	Abscess	[41]
*Fusobacterium necrophorum* subsp. *funduliforme*	Human, animal	Lemierre syndrome, postanginal sepsis, tonsillitis, peritonsillar abscess	[41]
*Fusobacterium perfoetens*	Dogs	Overweight	[42]

*Abbreviations*: APOs, adverse pregnancy outcomes; CRC, colorectal cancer; IBD, inflammatory bowel disease.

As research progresses, attention has shifted to the genetic and phenotypic heterogeneity of *F. nucleatum*, increasing the resolution of analysis to the subspecies level [43, 44, 45]. It has been reported that the *Fusobacterium* communities and *F. nucleatum* population distributions differ by body site. Saliva shows significantly higher *Fusobacterium* diversity than GIT samples. While no subspecies shows significant site‐specific enrichment, strain‐level analysis reveals distinct colonization patterns, suggesting selective pressure during translocation at the strain level [46]. The distribution of *F. nucleatum* populations in the gingiva significantly differed from that observed in stool of both CRC patients and healthy volunteers [47].

In dental plaque, *Fnp* and *Fnv* are the most common populations in both healthy controls and those with infections both in the sub‐ and supragingival plaque, whereas *Fnn* is among the least common [47]. In another review of literature, they reanalyzed publicly available datasets and found that *Fnv and Fna* dominated healthy plaque, *Fnp* peaked in gingivitis, and all subspecies elevated in periodontitis. Except for *Fnp*, the other three *F. nucleatum* subspecies occurred at similar frequencies in health, gingivitis, and periodontitis [48]. Though in Connolly's study [47] *F. nucleatum* populations were not detected in saliva, other studies detected *F. nucleatum* in the saliva of healthy individuals and patients with gingivitis, periodontitis, and gastric cancer (GC) by polymerase chain reaction (PCR), indicating that detection may be influenced by disease status [49, 50, 51]. While stool samples show no significant difference in *F. nucleatum* distribution between healthy and CRC cohorts, its detection frequency is higher in CRC. Absolute abundance of *F. nucleatum* populations is generally lower in stool than in gingiva, with the striking exception of a new clade of *Fna*, *Fna* C2, which is more prevalent in CRC‐associated stool samples than in gingival sites [14, 47]. These results are consistent with findings that *Fna* persists in the colon, whereas *Fnv* rarely colonizes the lower GIT [46].

Potential links between the distribution of *F. nucleatum* and demographics—sex, age, and weight—require further investigation, owing to the difficulty of separating them from other confounders [52]. The frequency of *F. nucleatum* detection are higher in males than in females, but sex‐specific analyses of *F. nucleatum* distribution in gingival and stool samples reveal no significant differences in overall detection frequencies among the sexes. At the subspecies level, however, *Fna* C2 and *Fnp* exhibit elevated abundance in male stool, particularly in Crohn's disease (CD) patients, suggesting sex‐related variation is predominantly disease dependent. Age‐stratified analyses demonstrate progressive enrichment of *F. nucleatum* in diseased cohorts, with *Fna* C2 dominant in both healthy and diseased individuals. *F. nucleatum* abundance increases with age in diseased patients, peaking in older males with CD, while remaining low across all ages in healthy cohorts [47]. As for weight, *F. nucleatum* is significantly more abundant in the mucosal colon microbiome of overweight/obese healthy controls compared with those of normal weight [53]. Thus, *F. nucleatum* population structure has robust implications for human health phenotypes. And demographic factors do not alter the rank order of *F. nucleatum* populations, underscoring the stability of subspecies hierarchy despite context‐dependent abundance shifts. These studies highlight that age, male sex, overweight, and disease synergistically amplify *F. nucleatum* colonization, with *Fna* C2 emerging as a potential biomarker of dysbiotic states.

Environmental modulators also influence *F. nucleatum* colonization. Smoking increases *F. nucleatum* abundance in both periodontally healthy and diseased populations, while chronic periodontitis patients with poorly controlled type‐2 diabetes harbor higher levels of *F. nucleatum* [19, 54, 55]. Dietary patterns are also associated with *F. nucleatum* infection [56, 57].

## 3 *F. nucleatum* in Health and Commensalism

### 3.1 Oral Niche and Role in Biofilm Ecology

The human oral microbiome evolves dynamically from birth, shaped by physiological changes, such as teeth eruption and teeth replacement and by environmental exposures like diet, life habits, and antibiotics use. *F. nucleatum* abundance in oral cavity increases in predentate infants with mothers who smoked, in children exposed to caregiver transmission, or early‐life antibiotics, and in individuals after 3 weeks of no oral hygiene, but shows no significant heritability in twin studies [58]. Oral microbial composition is niche specific, with distinct “normal” communities in saliva, supragingival plaque, subgingival plaque, and mucosa. The phylum Fusobacteriota is mainly detected in dental plaque [59].

**FIGURE 2:**
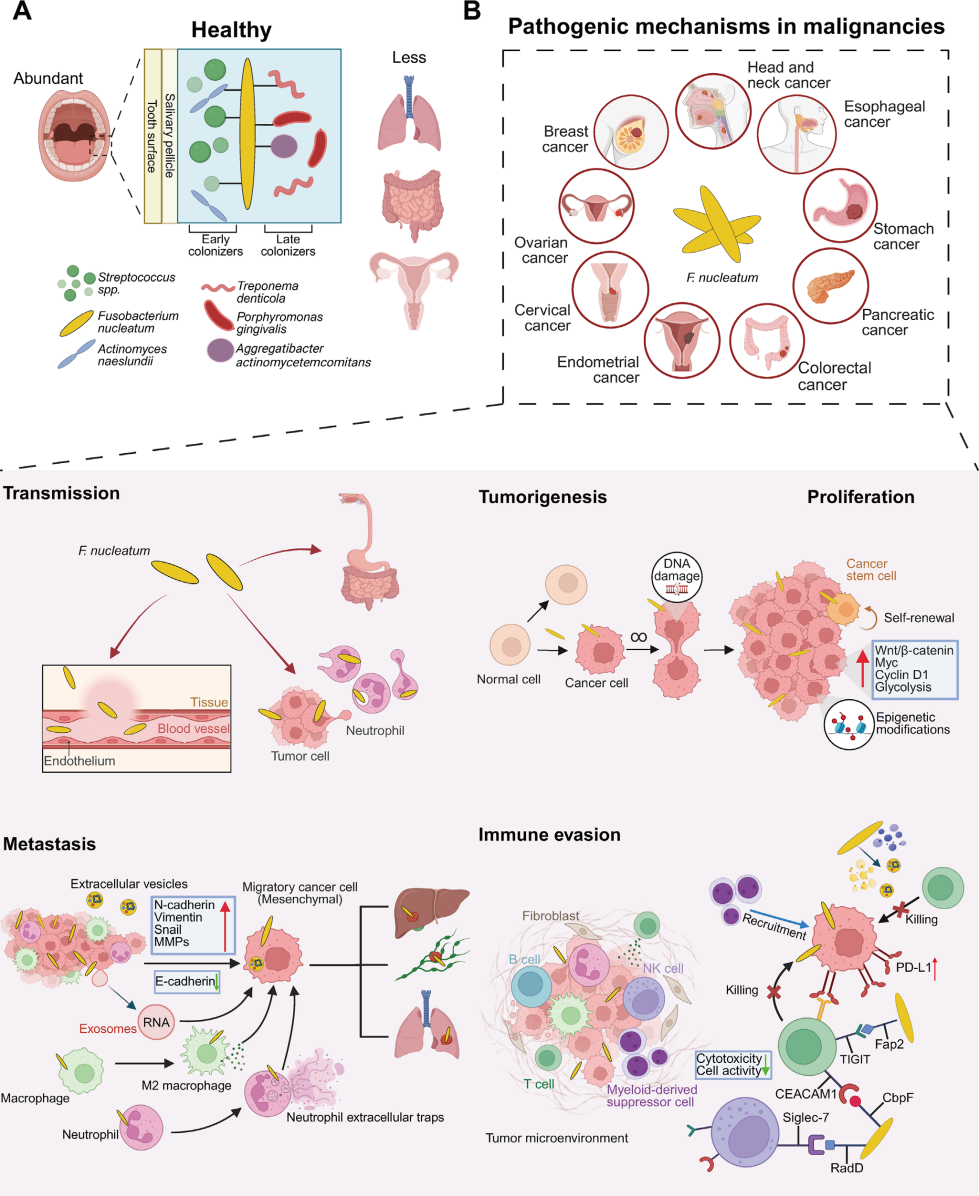
Schematic illustration of the dual role of *Fusobacterium nucleatum* (*F. nucleatum*) in health and cancer. (A) Under physiological conditions, *F. nucleatum* predominantly colonizes the oral cavity and is rarely detected at extra‐oral sites. As a commensal, it resides within dental plaque biofilms, where it facilitates coaggregation with other microorganisms and bridges early and late colonizers, thereby maintaining oral microbial architecture. (B) In pathological contexts, *F. nucleatum* is implicated in multiple malignancies, contributing to cancer initiation and progression through diverse mechanisms, including systemic dissemination, induction of tumorigenesis, promotion of cellular proliferation and metastasis, and facilitation of immune evasion. Created with BioRender.com. *Abbreviations*: MMPs, matrix metalloproteinases; TIGIT, T cell immunoglobulin and ITIM domain; CEACAM1, carcinoembryonic antigen related cell adhesion molecule 1.

Under physiological conditions, *F. nucleatum* as a commensal organism resides in the dental plaque biofilm, which supports local oral health by helping maintain pH balance and suppressing pathogen growth [60]. For microbial colonization, the oral cavity provides three distinct surfaces: teeth, mucosa, and preadhered bacteria via coaggregation. The distinctive rod shape and surface adhesion proteins of *F. nucleatum* facilitate coaggregation with other microorganisms, thereby establishing a physical connection between early colonizers, such as *Streptococcus*, and late colonizers, such as *Porphyromonas gingivalis* (*P. gingivalis*), *Aggregatibacter actinomycetemcomitans*, and so on, in dental plaque biofilm [61], as shown in Figure 2A.

The oral biofilm involves interspecies synergies and competition and can act like body tissues, maintaining homeostasis in the gingival epithelial barrier. Low‐level baseline inflammation and antimicrobial peptides production induced by commensal bacteria help preserve barrier integrity. In periodontal homeostasis, *F. nucleatum* employs its cell wall proteins—most notably the FadA adhesin—for attachment and invasion, while delivering lipopolysaccharide (LPS) selectively to oral epithelial cells. This targeted LPS delivery, coupled with the limited expression of toll‐like receptor (TLR) 4 within endosomal compartments, enables controlled interleukin (IL)‐8 release, thereby recruiting neutrophils exclusively to microbial challenge sites without triggering excessive inflammation. Healthy plaque microbiota counterbalances this process probably by secreting lipoteichoic acid, which antagonizes TLR4 hyperactivation, ensuring neutrophils surveil the junctional epithelium without tissue damage [62]. Besides, *F. nucleatum* activates protease‐activated receptor‐1 on oral epithelial cells to trigger IL‐8 signaling [62]. Exposed to *F. nucleatum*, human gingival epithelial cells can produce β‐defensin‐2 through TLR2. β‐defensin‐2 is a kind of antimicrobial peptides, which is an essential factor in enforcing the epithelial barrier by controlling the overgrowth of resident plaque bacteria and facilitating the healing processes of periodontal disease [62, 63, 64].

Metatranscriptomic studies demonstrate that *F. nucleatum* exhibited comparable abundance levels in healthy and periodontitis samples, yet its metabolic activity of converting lysine to butyrate is significantly upregulated under diseased conditions [65]. Transcriptome profiling further reveals that its in vivo gene expression profile in the periodontal pocket differs markedly from laboratory culture, with upregulation of hemin receptors, peptide transporters, and surface proteins [66]. A shift from commensal to pathogen may be triggered by oxidative stress [67], and exposure to stress hormones such as catecholamines and cortisol may also facilitate this transition [58].

### 3.2 Low‐Abundance Presence in Extra‐Oral Sites

In the gut, *F. nucleatum* is generally present at low abundance under healthy conditions but may contribute to pathogenic processes when gut microbiota becomes dysregulated [53, 68]. In the respiratory tract, *F. nucleatum* is rarely detected under normal conditions, though its presence has been linked to respiratory inflammation and disease states [69]. In the vagina, *F. nucleatum* is typically absent or found only in minimal amounts in healthy states, as its overgrowth is associated with bacterial vaginosis (BV) and an elevated risk of preterm birth [70] (Figure 2A).

### 3.3 Potential Beneficial Functions

Although numerous studies have established the promotive role of *F. nucleatum* in inflammation and cancer, emerging evidence suggests it may also exert beneficial effects. Velsko et al. [71] demonstrated that in a chronic oral monoinfection model, *F. nucleatum* reduced aortic plaque formation in ApoE^null^ mice, with significantly smaller plaque areas observed at 24 weeks compared with controls. This was accompanied by attenuated aortic inflammation, evidenced by decreased infiltration of F4/80^+^ macrophages and CD3^+^ T cells in both inner and outer vascular layers. Unlike other periodontal pathogens, *F. nucleatum* infection did not induce vascular endothelial dysfunction, indicated by unaltered nitric oxide levels, aligning with minimal plaque development. Despite elevating proatherogenic serum lipids and inflammatory markers, *F. nucleatum* concurrently enhanced anti‐inflammatory pathways and high‐density lipoprotein production, potentially counterbalancing atherogenic drivers.

The concentration of *F. nucleatum* appears to influence its biological effects. Based on Heusler et al. [72]’s in vitro study, low concentrations of *F. nucleatum* promotes early pregnancy development by enhancing trophoblast invasion in HTR8/SVneo cells and stimulating the secretion of key mediators like chemokines and matrix metalloproteinases (MMPs). These proinvasive effects are mediated partly through TLR4 signaling and are independent of LPS at low bacterial loads. In contrast, higher bacterial concentrations impair trophoblast function by reducing cell viability, hindering migration, and altering the cell cycle, highlighting a concentration‐dependent dual role. Similarly, Einenkel et al. [73] reported that minimal *F. nucleatum* exposure induced hypoxia‐inducible factor (HIF) activation and VEGF‐A secretion in regulatory macrophages, thereby enhancing trophoblast tube formation—a critical process for placental vascularization. Controlled *F. nucleatum* presence may thus mimic physiological bacterial residues, promoting immune balance conducive to implantation and early pregnancy development without eliciting destructive inflammation.

In oncology, *F. nucleatum‐*driven programmed death ligand 1 (PD‐L1) overexpression in tumor cells promotes immune evasion but may paradoxically sensitize tumors to immune checkpoint blockade therapy [74]. Single‐cell RNA sequencing analyses indicate that *F. nucleatum* can remodel the TME, increasing immune cell infiltration and modulating cell–cell interactions, thereby enhancing CRC responsiveness to PD‐L1 blockade [75]. Mechanistically, *F. nucleatum* activates STING signaling, leading to PD‐L1 upregulation and recruitment of interferon (IFN)‐γ^+^ CD8^+^ tumor‐infiltrating lymphocytes, which augment tumor sensitivity to PD‐L1 blockade and improve survival outcomes [76]. Similarly Wang et al. [77] revealed that high levels of intratumoral *F. nucleatum* correlated with favorable responses to anti‐programmed death 1 (PD‐1) therapy in microsatellite stable CRC patients. This effect was mediated by butyric acid, which inhibited histone deacetylase 3/8 in CD8^+^ T cells, increasing acetylation and expression of the *TBX21* gene. And TBX21 in turn repressed PD‐1 expression, reduced CD8^+^ T cell exhaustion, enhanced the effector function, and potentiated anti‐PD‐1 efficacy.

## 4 Pathogenic Mechanisms of *F. nucleatum*

### 4.1 Adhesion, Colonization, and Invasion

Bacterial interactions with host epithelial surfaces are fundamental determinants of infection. Adhesion to epithelial cells provides the essential initial foothold for microbial persistence, enabling subsequent colonization. Once established, invasion represents a critical pathogenic strategy, allowing microbes to evade host immune surveillance and penetrate into deeper tissues. Attachment and invasion are hallmarks of *F. nucleatum*, mediated by the specific interaction between its surface adhesins like FadA, Fap2, and RadD and corresponding host receptors, via a “zipping” mechanism that maintains close contact with the host cell membrane [6, 78, 79, 80, 81]. This process requires active participation of host cell machinery, including actin polymerization, microtubule function, signal transduction, protein synthesis, and energy metabolism. While internalized *F. nucleatum* is typically trafficked to endocytic degradation pathways without causing cytopathic effects, successful invasion is required for triggering potent proinflammatory responses that drive tissue destruction, particularly in contexts where epithelial barriers are compromised [6, 64].

*F. nucleatum—*though primarily an oral commensal—has been detected in multiple extra‐oral sites, particularly under pathological conditions. However, its origin and systemic dissemination routes require further investigation. Studies in CRC provide the most compelling evidence for *F. nucleatum* involvement in disease, and insights from these studies may inform research into other pathologies. Possible transmission routes and pathogenesis of *F. nucleatum* in malignancies are illustrated in Figure 2B. Current evidence suggests several potential dissemination routes: (i) gastrointestinal translocation, (ii) hematogenous spread, (iii) comigration with tumor or immune cells, and (iv) direct mucosal contact transmission.

- *Gastrointestinal translocation*: As oral cavity is a reservoir of *F. nucleatum*, oral–gut migration through swallowing is plausible—supported by the high salivary abundance in GC patients and its detection in nasopharyngeal [51, 82]. It is reported that both *F. nucleatum* detection rates in cancer samples and its DNA amount in the normal epithelium show decreasing trends as the site moved from upper GIT toward the anorectal side, which may be explained by the reduced viability and activity of *F. nucleatum* in long exposure to a low pH environment [83].
- *Hematogenous spread*: Interestingly, the abundance of *F. nucleatum* in normal and tumor colorectal tissues are respectively higher than in the corresponding gastric tissues, despite the anatomical distance [84]. A portion of *F. nucleatum* detected in CRC tissue originates from the oral cavity and is more likely to colonize the GIT via the bloodstream rather than through oral gavage, even if there is a disadvantage in quantity [85, 86]. Oral *F. nucleatum* may enter the bloodstream during periodontal bleeding caused by disease or dental procedures, leading to bacteremia. Once in circulation, its surface adhesin FadA could bind to vascular E‐cadherin, compromising tight junctions to increase permeability and facilitate transmigration to tissues with abundant blood supply [87].
- *Comigration with tumor or immune cells*: *F. nucleatum* may be transported along with metastasizing tumor cells to distant sites after its adhesion and invasion. It can also disseminate within neutrophil by surviving phagocytosis, reducing reactive oxygen species (ROS) accumulation, and producing hydrogen sulfide (H_2_S) [88, 89]. This “Trojan horse” mechanism parallels observations of *P. gingivalis* translocation within dendritic cells from oral cavities to aortic walls [90, 91].
- *Direct mucosal contact transmission*: Detection of oral commensals, including *F. nucleatum*, in the breast and vaginal microbiota suggests possible oral–mammary or oral–vaginal contact transmission [92, 93].

The pH value appears to be a critical determinant of *F. nucleatum* survival during gastrointestinal transmission. Proton pump inhibitors increase more *F. nucleatum* migration from the oral cavity to the gut compared with histamine 2 receptor antagonists since proton pump inhibitors can affect the diversity of the gastric microbiome either by directly targeting the bacterial and fungal proton pumps or by raising intragastric pH, disrupting the normal gastric microenvironment and allowing more passage and survival of microbiota from the oral cavity [94]. Similarly, elevated vaginal pH promotes *F. nucleatum* colonization through strong Fap2–Gal–glycan binding [95]. Acid tolerance mechanisms further enhance its dissemination potential. Amyloid‐like FadA which *F. nucleatum* produced under stress and diseased conditions confers acid tolerance, aiding survival during gastrointestinal transit and within the TME [78]. Additionally, high content of erucic acid [C22:1(n9)] in cell membrane of *F. nucleatum*, which is regulated by the enoyl‐CoA hydratase‐related protein FnFabM, contributes to acid resistance, facilitating intestinal colonization [96].

### 4.2 Modulation of Host Signaling Pathways

Cellular signaling pathways transduce extracellular cues into precise intracellular responses that coordinate essential physiological processes. Dysregulation of these pathways underlies disease pathologies, driving persistent inflammation, uncontrolled cell proliferation, or impaired cell death programs. As an opportunistic pathogen, *F. nucleatum* perturbs multiple host signaling cascades, most prominently by activating the Wnt/β‐catenin pathway to promote cell proliferation and modulating the nuclear factor‐kappa B (NF‐κB) pathway to regulate inflammation.

#### 4.2.1 Wnt/β‐catenin and Proliferation

Wnt/β‐catenin signaling represents a fundamental regulator of epithelial homeostasis and cell proliferation. *F. nucleatum* directly hijacks this pathway via its surface adhesin FadA, which binds to host E‐cadherin, and initiates a proproliferative signaling cascade critical for CRC progression. This interaction disrupts the E‐cadherin/β‐catenin complex via Annexin A1, liberating β‐catenin to translocate into the nucleus, where it activates transcription factors lymphoid enhancer factor /T cell factor and upregulates oncogenes *MYC* and *Cyclin D1—*key drivers of uncontrolled cell proliferation [6, 87, 97]. *F. nucleatum* also amplifies Wnt/β‐catenin signaling by inducing overexpression of CDK5, which reinforces downstream proliferative targets [98]. Notably, *F. nucleatum* enrichment in precancerous lesions correlates with nuclear β‐catenin accumulation and activation of the β‐catenin/REG1α axis, where REG1α promotes CRC cell proliferation through β‐catenin/MYC signaling [99]. Collectively, by FadA–E‐cadherin involvement and subsequent dysregulation of β‐catenin‐dependent transcription, *F. nucleatum* acts as a direct microbial driver of pathological host cell proliferation in colorectal carcinogenesis.

#### 4.2.2 NF‐κB and Inflammation

NF‐κB functions as a central transcriptional regulator of inflammation, orchestrating the expression of proinflammatory cytokines and mediating both acute and chronic inflammatory responses. Under physiological conditions, low‐level inflammation in epithelial cells maintains homeostasis, but disruption of this equilibrium enables *F. nucleatum* to trigger persistent inflammation, fueling disease initiation and progression. In the oral cavity, *F. nucleatum* invasion of human gingival fibroblasts robustly activates NF‐κB, driving sustained cytokine release. This robust response reflects the absence of tolerance to bacterial stimulation in gingival fibroblasts, which continuously produce high‐level inflammatory cytokines upon exogenous challenge [100]. Chronic inflammation is a critical factor in carcinogenesis, as exemplified by *Helicobacter pylori* (*H. pylori*), and *F. nucleatum* also exhibits proinflammatory properties distinct from those of *H. pylori* in GC. Niikura et al. [101] found that *F. nucleatum* stimulated gastric epithelial cells resulting in a robust activation of the NF‐κB and serum response element pathways. Geneset enrichment analysis revealed enrichment of inflammatory response, apoptosis, and metabolic pathways in GC cell line cocultured with *F. nucleatum*, which were not significantly altered by *H. pylori*. Mechanistically, *F. nucleatum* enhanced ROS production and engaged the TLR4‐NF‐κB axis via its outer membrane LPS, promoting secretion of proinflammatory cytokines including IL‐6, IL‐8, and tumor necrosis factor (TNF)‐α.

In macrophages, *F. nucleatum* activates NF‐κB to exert bidirectional immunomodulatory effects. High bacterial loads drive classical NF‐κB hyperactivation, upregulating proinflammatory cytokines, antigen‐presenting molecules, and tissue‐destructive responses—exemplified by impaired trophoblast functions in pregnancy and foam cell formation in atherosclerosis [4, 73]. Conversely, in TME and tolerogenic settings, *F. nucleatum* exploits NF‐κB pathway to polarize macrophages toward M2 phenotype, which exhibit superficially anti‐inflammatory properties but ultimately facilitate immune evasion in CRC or support angiogenic reprogramming through VEGF [73, 102]. Critically, low‐dose *F. nucleatum* in decidual M2c macrophages diverts NF‐κB signaling toward HIF‐1α stabilization, suppressing destructive inflammation while enhancing VEGF‐A‐mediated trophoblast tube formation [73]. Thus, *F. nucleatum*‐NF‐κB crosstalk either amplifies inflammation or orchestrates immune tolerance, with M2 polarization serving as a pivot between pathological immunosuppression and physiological tissue adaptation.

Additionally, extracellular vesicles (EVs) derived from *F. nucleatum*, especially outer membrane vesicles (OMVs), contribute to NF‐κB‐mediated inflammation in periodontitis, further amplifying host immune responses [103, 104].

### 4.3 Induction of Genomic Instability

Genomic instability characterized by DNA damage, impaired repair mechanisms, and aberrant cell cycle progression is a hallmark of aging and a fundamental driver in cancer development. *F. nucleatum* accelerates tumor progression by inducing genomic instability through direct DNA damage, interference with repair mechanisms, and epigenetic reprogramming. *F. nucleatum* infection directly triggers DNA double‐strand breaks (DSBs), as evidenced by upregulated γ‐H2AX in oral cancer and CRC cells, mediated through both physical interaction and secretion of genotoxic metabolites [105, 106, 107]. The virulence factor FadA upregulates the DNA damage kinase Chk2 and induces S‐phase cell cycle arrest, which favors mutagenic proliferation [106]. Concurrently, *F. nucleatum* further exacerbates genomic chaos by suppressing the DSBs repair protein Ku70, which inactivates the tumor suppressor p53 and downregulates the cell‐cycle inhibitor p27, thereby crippling critical DNA damage response pathways [105]. *F. nucleatum* colonization correlates with high microsatellite instability (MSI) and poor prognosis, which is also mediated by bacterial metabolites. N‐acetylmuramic acid and mesaconic acid may be two major genotoxins secreted by *F. nucleatum*, inducing DSBs and promoting mutational accumulation during carcinogenesis [101]. In addition, *F. nucleatum* releases low‐molecular‐weight, heat‐stable mutagens—DL‐homocystine and allantoic acid—that independently induce γ‐H2AX foci and DSBs in colonic epithelial cells [108]. Another metabolite, H_2_S, has been demonstrated to generate ROS, leading to DNA damage and the occurrence of single‐nucleotide mutations [109, 110].

Beyond direct DNA damage, *F. nucleatum* may play a role in epigenetic modifications, including promoter DNA and RNA methylation and histone methylation and acetylation. High level of *Fusobacterium* in cancer tissues and *F. nucleatum‐*positive cancer tissues are associated with certain epigenetic alterations [84, 111, 112]. For example, the enrichment of *F. nucleatum* in head and neck squamous cell carcinoma correlates with hypermethylation of tumor suppressor genes such as *LATEXIN* and *SMARCA2*, suggesting that *F. nucleatum* infection may promote cell proliferation through epigenetic silencing [113].

### 4.4 Promotion of Migration, Invasion, and Metastasis

*F. nucleatum* has been implicated in the enhancement of migration and invasion of various malignant cells through direct tumor cell modulation, EVs‐mediated communication, and immune cell reprogramming. Infected cancer cells exhibit enhanced motility and invasiveness via promoting MMPs expression, inducing epithelial–mesenchymal transition (EMT) and disrupting metabolism [83, 97, 114, 115, 116, 117]. These processes disrupt epithelial integrity and degrade extracellular matrix barriers, facilitating local invasion and distant dissemination. In addition, *F. nucleatum* alters exosomal cargo in infected CRC cells and GC cells, packaging them with noncoding RNAs and chemokines. These exosomes, when taken up by uninfected tumor cells, activate promigratory signaling cascades, thereby enhancing in vitro invasion and promoting metastasis in vivo [118, 119]. Beyond direct tumor cell effects, *F. nucleatum* shapes the metastatic niche by recruiting and reprogramming macrophages and neutrophils toward protumoral phenotypes [74, 102, 120]. Tumor‐associated macrophages polarized by *F. nucleatum* may support metastasis by inhibiting tumoricidal immune response, initiating angiogenesis, and activating matrix remodeling [121]. Neutrophils, activated through TLR4–ROS and NOD1/2 signaling by *F. nucleatum*, form abundant neutrophil extracellular traps. These neutrophil extracellular traps indirectly promote metastasis by inducing EMT in tumor cells, enhancing MMPs expression and physically trapping circulating tumor cells to facilitate dissemination [122].

### 4.5 Modulation of Tumor Immune Microenvironment

Beyond its direct interaction with tumor cells, *F. nucleatum* profoundly remodels the tumor immune microenvironment (TIME) by suppressing antitumor immunity, amplifying proinflammatory signaling, and fostering an immunosuppressive niche. *F. nucleatum* recruits myeloid‐derived suppressor cells into the TME, significantly suppressing T cell activity [123, 124]. It also directly engages inhibitory receptors presenting on tumor‐infiltrating T lymphocytes and natural killer (NK) cells, inhibiting T cell activity and NK cell cytotoxicity, which serve to protect both the bacteria itself and adjacent tumor cells [125, 126, 127]. In esophageal squamous cell carcinoma (ESCC), *F. nucleatum* enrichment is associated with an immunosuppressive phenotype, including increased regulatory T cells and elevated expression of inhibitory receptors on CD8^+^ T cells [128, 129]. In murine Atp4b‐Il1b mice models—characterized by gastric inflammation, atrophy, and epithelial hyperproliferation independent of *H. pylori* infection—*F. nucleatum* treatment causes more robust infiltration of immune cells, particularly F4/80^+^ macrophages and CD11c^+^ dendritic cells. Lymphoid follicles containing CD3^+^ T lymphocytes, CD45R^+^, and/or CD19^+^ B lymphocytes are more abundant, alongside a rise in the population of T lymphocytes, including both CD4^+^ and CD8^+^ lineage, within the lamina propria [101]. As mentioned above, *F. nucleatum* also mobilizes macrophages and neutrophils, further reinforcing a protumoral TIME.

In addition to cell–cell interactions, *F. nucleatum* is capable of modulating immunity through its metabolites. Its metabolite 3‐indolepropionic acid activates AhR in macrophages, inducing M2 polarization and immunosuppressive programming that promote CRC progression [130]. Its colonization in murine models increases the level of immunomodulatory short‐chain fatty acids (SCFAs) in the colon. These SCFAs elicit numerous changes in different types of immune cells and influence the production of cytokines and chemokines by intestinal epithelial cells, which can activate and attract immune cells [131, 132, 133].

### 4.6 Contribution to Therapy Resistance

Chemotherapy and immunotherapy are important treatments for patients with advanced or recurrent malignancies. Existing studies indicate that high intratumoral abundance of *F. nucleatum* is strongly associated with chemoresistance and reduced immunotherapy efficacy across multiple cancer types. Clinical studies have shown that elevated *F. nucleatum* burdens diminish the efficacy of 5‐fluorouracil‐based adjuvant chemotherapy in advanced CRC patients after surgery and reduce the benefit of neoadjuvant chemotherapy in ESCC patients. Mechanistically, *F. nucleatum*‐mediated chemoresistance primarily involves modulation of programmed cell death pathways, including modulating autophagy, inhibiting pyroptosis, and inhibiting ferroptosis [134, 135, 136, 137, 138]. Additionally, the exosomes from *F. nucleatum*‐infected cells containing circRNA confer oxaliplatin or 5‐fluorouracil resistance by alleviating endoplasmic reticulum stress [139]. In ESCC, *F. nucleatum* invades chemotherapy‐induced senescent cells, enhancing the protumorigenic senescence‐associated secretory phenotype (SASP). This *F. nucleatum*‐driven SASP amplification in turn promotes chemoresistance in ESCC, correlating with poor prognosis [140].

Beyond promoting chemoresistance, *F. nucleatum* can also impair the efficacy of immunotherapy. The PD‐1/PD‐L1 immune checkpoint blockade therapy aims to restore T cell‐mediated tumor immunity by blocking PD‐1/PD‐L1 signaling; however, *F. nucleatum* counteracts this in multiple cancers. Chen et al. [89] demonstrated that *F. nucleatum* specifically induced PD‐L1 overexpression in phagocytes, generating immunosuppressive CX3CR1^+^PD‐L1^+^ subsets that trafficked to tumors, thereby reducing CD8^+^ T cell infiltration and enhancing metastasis, ultimately diminishing αPD‐L1 therapy efficacy. Zhang et al. [74] found *F. nucleatum* invasion of tumor cells activated IL17/NF‐κB/RelB signaling, promoting the recruitment of tumor‐associated neutrophils and the differentiation of PD‐L1^+^ tumor‐associated neutrophils. These neutrophils differentiated into a protumoral subtype, elevating PD‐L1 expression to facilitate immune evasion. Such immunosuppressive effects have been observed not only in CRC, but also in breast cancer (BC) and ESCC, where *F. nucleatum* suppresses T cell function and upregulates PD‐L1, thereby impairing immunotherapy efficacy [141, 142, 143].

Moreover, *F. nucleatum‐*derived metabolites add another layer to immune escape. For example, succinic acid produced by *F. nucleatum* activates the succinic acid receptor SUNCR1–HIF‐1a–EZH2 axis in tumor cells. This activation subsequently inhibits the cGAS–IFN‐β pathway, leading to a reduction in the secretion of T helper 1‐type chemokines, specifically C‐C motif chemokine ligand (CCL) 5 and C‐X‐C motif chemokine ligand (CXCL) 10. Consequently, this downregulation affects the recruitment and activation functions of CD8^+^ T cells, thereby impairing the antitumor efficacy of PD‐1 blockade in CRC [144]. Likewise, the conserved bacterial metabolite ADP‐heptose activates ALPK1, differentially regulating cancer‐related pathways and significantly increasing PD‐L1 expression in an ALPK1‐dependent manner [145]. *F. nucleatum* derived OMVs can also impair T cell function and blunt immunotherapy response through TDO2/AHR activation of tumour‐associated macrophages [146].

## 5 *F.nucleatum* in Nonmalignant Inflammatory Diseases

### 5.1 Oral Inflammatory Diseases

**FIGURE 3:**
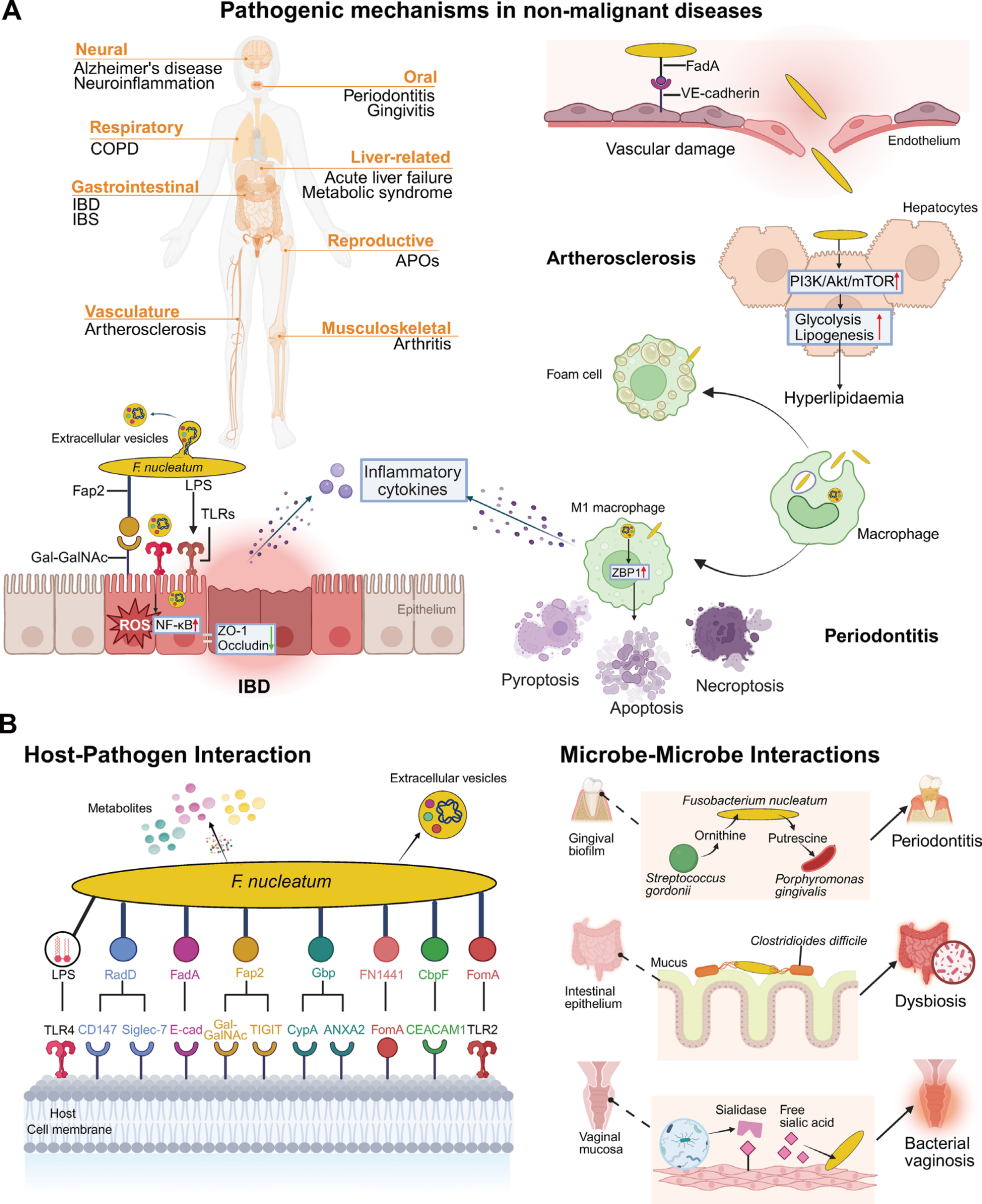
Pathogenic mechanisms of *Fusobacterium nucleatum* (*F. nucleatum*) across nonmalignant diseases. (A) In pathological states, *F. nucleatum* is associated with a broad range of nonmalignant diseases, including oral inflammatory diseases and extra‐oral diseases such as periodontitis, atherosclerosis, and inflammatory bowel disease (IBD). In atherosclerosis, it compromises endothelial integrity, promoting hepatic lipogenesis, and drives foam cell formation. In periodontitis, it induces proinflammatory PANoptosis via ZBP1, amplifying cytokines release. In IBD, it impairs the epithelial barrier integrity by decreasing tight junction proteins and induces robust inflammatory cytokines expression. (B) *F. nucleatum* perturbs ecological homeostasis and exerts pathogenic effects through both direct and indirect host interactions, as well as microbe–microbe interactions involving metabolic cross‐feeding and physical coaggregation. Created with BioRender.com. *Abbreviations*: COPD, chronic obstructive pulmonary disease; IBS, irritable bowel syndrome; APOs, adverse pregnancy outcomes; VE‐cadherin, vascular E‐cadherin; LPS, lipopolysaccharide; TLRs, toll‐like receptors; ROS, reactive oxygen species; E‐cad, E‐cadherin; Gal‐GalNAc, d‐galactose‐β (1‐3)‐N‐acetyl‐d‐galactosamine; TIGIT, T cell immunoglobulin and ITIM domain; ANXA2, Annexin A2; CEACAM1, carcinoembryonic antigen related cell adhesion molecule 1.

*F. nucleatum* is a common constituent of the sub‐ and supragingival biofilms in both diseased and healthy individuals, functioning as a bridging organism through the coordinated expression of multiple adhesins [87, 147, 148, 149, 150, 151]. Clinical studies have shown its strong association with various forms of periodontal diseases, including gingivitis, periodontitis, and endodontic infections [49, 152, 153, 154, 155, 156, 157, 158, 159, 160, 161, 162, 163, 164, 165]. Notably, its prevalence escalates with disease severity, inflammatory progression, and periodontal pocket depth [152, 157, 166]. Higher *F. nucleatum* abundance is observed in gingivitis and periodontitis patients compared with healthy controls, and its serum antibody levels correlate positively with disease activity [49, 50, 167]. Experimental studies further substantiate its etiological role in periodontal infections. In murine models, monoinfection with *F. nucleatum* induces alveolar bone resorption or abscess formation [168]. Moreover, synergistic virulence is observed in coinfection with other oral pathogens, leading to amplified bone loss, abscess severity, or host mortality [169, 170, 171, 172, 173]. Mechanistically, *F. nucleatum* orchestrates periodontitis pathogenesis by triggering host hyperimmunity in periodontal stem cells, while concurrently inhibiting osteogenic differentiation via surface adhesins [103, 174, 175]. Additionally, *F. nucleatum* induces proinflammatory PANoptosis via ZBP1 sensing in periapical tissues, amplifying cytokines release and alveolar bone destruction [176] (Figure 3A).

In previous studies, metagenomic sequencing targeting the 16S rRNA gene, while widely used for characterizing microbiota, has lacked the resolution necessary to differentiate the four *F. nucleatum* subspecies [65]. Consequently, most investigations have historically treated all *F. nucleatum* subspecies as functionally equivalent in both experimental and clinical contexts, potentially obscuring subspecies‐specific roles in oral ecology and disease pathogenesis. This methodological limitation impedes dissecting subspecies‐level heterogeneity, necessitating advanced genomic tools. Recent advances have begun to challenge this assumption, proposing that distinct subspecies of *F. nucleatum* exhibit niche‐specific colonization patterns within the oral cavity, with some (e.g., *Fnp*, *Fnv*) preferentially associating with health‐associated biofilms and others (e.g., *Fnn*, *Fna*) being enriched in disease‐associated inflammatory milieus, reflecting their divergent functional adaptation to host–microbe interactions [7, 17, 177, 178]. Underlying metabolic distinctions may, at least in part, account for these patterns. The enrichment of *Fna* in odontogenic abscesses appears linked to its unique genomic capacity to encode the high‐affinity ferrous iron‐transport system FeoA/B, which is critical for virulence in anaerobic/hypoxic abscess environments where reduced ferrous iron is abundant. In contrast, the enhanced metabolic capacities of *Fnp*, including multiple amino acid biosynthesis pathways (e.g., methionine, cysteine) and the molybdenum transporter ModF, confer a competitive fitness advantage within dental plaque [7]. These studies support that the four species should be considered distinct entities in clinical and laboratory investigations, owing to their phenotypic and genotypic differences.

### 5.2 Extra‐Oral Inflammatory Diseases

#### 5.2.1 Atherosclerosis

It is well established that periodontitis is an important risk factor for atherosclerosis [179, 180, 181]. Given that *F. nucleatum* is recognized as one of the keystone pathogens in periodontitis, researchers have sought to elucidate its potential contribution to vascular disease pathogenesis. Accumulating evidence indicates its role in cardiovascular diseases, with its DNA frequently detected within atherosclerotic plaques. Notably, this pathogen has been ranked among the most prevalent microorganisms found in both atherosclerotic lesions and ruptured cerebral aneurysms, highlighting its potential involvement in vascular wall injury and plaque destabilization [182, 183, 184].

Mechanistically, *F. nucleatum* appears to promote atherogenesis by acting at multiple stages of the disease cascade, from initiating endothelial injury to driving lipid accumulation (Figure 3A). First, *F. nucleatum* can directly causes endothelial cell damages. Its infection downregulates platelet endothelial cell adhesion molecule‐1 expression and triggers endothelial cell apoptosis, thereby compromising vascular integrity [87, 185, 186]. In addition, its virulence‐associated heat‐shock protein GroEL upregulates proinflammatory chemokines and adhesion molecules in human microvascular endothelial cells, potentiating endothelial dysfunction and leukocyte recruitment during early atherogenesis [187]. Once endothelial integrity is compromised, *F. nucleatum* disrupts macrophage lipid homeostasis via activation of the PI3K–AKT/MAPK/NF‐κB signaling axis, enhancing cholesterol uptake, impairing lipid efflux, and promoting intracellular lipid deposition. This cascade accelerates foam cell formation, a hallmark of early plaque development [4]. Beyond local vascular effects, *F. nucleatum* contributes to systemic hyperlipidemia by reprogramming hepatic lipid metabolism. Zhou et al. [188] identified it as a plausible link between periodontitis and increased hepatic lipogenesis, mediated by PI3K–Akt–mTOR activation, which promoted glycolysis and lipid synthesis in hepatocytes. This metabolic shift may further fuel circulating lipid supply for plaque growth.

#### 5.2.2 Arthritis

Although *F. nucleatum* has been sporadically isolated from cases of septic arthritis [189, 190, 191, 192, 193], most research has focused on its potential involvement in rheumatoid arthritis (RA), a chronic autoimmune disorder. Epidemiological studies have demonstrated a strong association between periodontitis severity and RA disease activity, supported by the high prevalence of periodontitis in RA patients and a dose–response relationship, leading to the hypothesis that oral microbiota—including *F. nucleatum—*may serve as a mechanistic link between the two diseases [194, 195, 196, 197, 198, 199]. Notably, periodontal therapy has been shown to improve RA clinical outcomes, further supporting this connection [200]. Clinical investigation further reveals that enrichment of *F. nucleatum* in the oral microbiome of RA patients—particularly those seropositive for anti‐cyclic citrullinated peptide antibodies—may synergize with severe periodontitis to exacerbate RA progression [201]. Mechanistically, *F. nucleatum*‐derived OMVs can deliver the adhesin FadA into joint synovium, where it activates Rab5a–YB‐1 signaling in synovial macrophages, thereby triggering synovial inflammation [199].

#### 5.2.3 Inflammatory Bowel Disease

IBD, which primarily includes ulcerative colitis and CD, has been increasingly linked to *F. nucleatum* in clinical and mechanistic studies. Clinical evidence indicates that *F. nucleatum* is enriched in ulcerative colitis and CD patients, correlating with disease activity, and has been incorporated into bacterial marker panels for CD diagnosis [5, 202, 203, 204]. Strains isolated from IBD lesions display higher invasiveness compared with those from healthy controls, which may be attributed to distinct subspecies or strain variation [205].

In both dextran sulfate sodium‐induced murine colitis models and in vitro experiments, *F. nucleatum* exacerbates colitis in direct and indirect ways. It impairs the epithelial barrier integrity by decreasing tight junction proteins, induces robust inflammatory cytokine expression, triggers endoplasmic reticulum stress, accelerates cellular senescence, and promotes multiple forms of cell death [202, 206, 207, 208, 209]. Multiple mechanisms involve in *F. nucleatum*‐induced cell death, including excessive autophagy, apoptosis, pyroptosis, and ferroptosis [204, 208, 210, 211, 212]. Beyond its interactions with epithelial cells, *F. nucleatum* exacerbated intestinal inflammation by blunting the therapeutic efficacy of anti‐TNF drugs and modulating immune cell function, notably by driving proinflammatory M1 macrophage polarization [213, 214].

Beyond host–pathogen interactions, *F. nucleatum* reshapes gut microbiota composition in ways that  exacerbate intestinal inflammation. It disrupts microbial diversity and stability by reducing beneficial taxa and enriching opportunistic pathogen, thereby impeding mucosa remission [215, 216, 217].

Psychosocial stress, a known exacerbating factor in IBD [218], may potentiate *F. nucleatum*‐driven pathology. Stress‐related norepinephrine directly binds to the quorum‐sensing regulator QseC of *F. nucleatum*, enhancing its pathogenicity and further aggravating colitis [219].

## 6 *F. nucleatum* in Malignancies

### 6.1 Colorectal Cancer

The potential link between *F. nucleatum* and CRC, first reported in 2011, has since spurred extensive research into its mechanistic and clinical relevance [220, 221]. In 2013, Rubinstein et al. [6] and Kostic et al. [123] demonstrated, in vitro and in vivo, that *F. nucleatum* promoted colorectal carcinogenesis by regulating the inflammatory and oncogenic signaling through its unique adhesion FadA, while simultaneously reshaping the TIME to favor tumor growth. Multiple studies conducted across Asia, Europe, and the Americas, supported by multiple meta‐analyses, have consistently shown significant enrichment of *F. nucleatum* in CRC tissues compared with adjacent normal tissue and healthy controls, as well as in colorectal polyps and fecal samples from CRC patients. Tumor tissues exhibit 2‐ to 415‐fold increase in bacterial load depending on the patient cohort and detection methods [222, 223].

CRC develops through initiation, promotion, progression, and metastasis stages over decades, primarily via three molecular pathways: the chromosomal instability‐driven adenoma–carcinoma sequence, the serrated pathway involving CpG island methylator phenotype (CIMP), and the inflammation‐driven pathway [224]. *F. nucleatum* mainly appears implicated in the serrated pathway. It colonizes sessile serrated adenomas/polyps in over 50% of lesions and may promote lesion proliferation by inducing β‐catenin nuclear translocation and upregulating REGIα expression [99]. Simultaneously, *F. nucleatum* exhibits strong molecular associations with high MSI, a phenotype mechanistically linked to CIMP. High *F. nucleatum* load correlates with a fivefold increased risk of MSI‐high CRC, independent of CIMP or BRAF status, and is mechanistically associated with epigenetic silencing of *MLH1*/*CDKN2A*, along with MSI‐related mutational signatures [225, 226]. Moderate bacterial burdens, in contrast, are linked to inflammation‐driven microsatellite alterations [107]. Additionally, multiple studies have demonstrated that *F. nucleatum* and its metabolites can induce DNA damage, thereby accelerating tumor evolution [106, 107, 108, 227]. In adenoma–carcinoma pathway, *F. nucleatum* abundance exhibits a stepwise escalation through the chromosomal instability pathway, although discrepancies exist in studies using fecal samples [225]. In the inflammatory pathway, while less defined, *F. nucleatum* has been shown to synergize with colitis‐induced injury to accelerate EMT via EGFR/AKT/ERK signaling in colitis‐associated cancer [228]. Metagenomics further confirms a progressive increase in *F. nucleatum* abundance from intramucosal carcinomas to advanced‐stages tumors [229]. Regarding anatomic location, earlier studies debated whether *F. nucleatum* exhibited a proximal‐distal colonic preference, yet a recent large‐scale study demonstrated consistent enrichment across all CRC tumor locations, suggesting no significant locational preference [230].

Besides its role in initiation, *F. nucleatum* also contributes to the subsequent stages of CRC progression. Critically, high intratumoral *F. nucleatum* abundance correlates with advanced clinicopathological features, including larger tumor size, poorer histological differentiation, lymph node and distant metastases, deeper invasion, and higher tumor stage, as well as poorer clinical outcomes such as reduced disease‐free survival and increased recurrence rates, particularly evident in Asian cohorts [223]. These epidemiological associations align with mechanistic and preclinical evidence, which collectively implicate *F. nucleatum* in driving CRC progression through sustained cancer cell proliferation, facilitation of metastatic spread, suppression of antitumor immunity, induction of proinflammatory cytokines, and promotion of therapeutic resistance [231].

### 6.2 Other Gastrointestinal Cancers

With the advent of high‐throughput sequencing technologies, increasing evidence has emerged linking local microbiota to gastrointestinal cancers (GICs), including GC. Historically, the stomach was considered a sterile organ, inhospitable to microbial colonization due to its highly acidic environment. This paradigm shifted dramatically following the discovery of *H. pylori*, which demonstrated that certain microorganism can persistently colonize gastric mucosa and elicit a chronic inflammatory response that may last for decades. Infected individuals face an estimated lifetime risk of 10–20% for developing peptic ulcer disease and 1–2% for progressing to GC, particularly in populations with specific host‐genetic susceptibilities. Owing to its established carcinogenic potential, *H. pylori* has been classified as a Group I carcinogen by the International Agency for Research on Cancer (IARC) under the World Health Organization framework [232, 233, 234, 235].

Although numerous studies have established associations between GICs and alterations in microbial signatures, no bacterial species beyond *H. pylori* has yet been conclusively classified as a carcinogen [235]. However, since the intensified investigation of *F. nucleatum* in CRC beginning in 2011 [220, 221], this organism has increasingly emerged as a candidate oncobacterium, implicated not only in CRC but also in esophageal, gastric, and pancreatic cancers. Yamamura et al. [236] first revealed that *F. nucleatum* detection in esophageal cancer (EC) tissues correlated with advanced tumor stage, reduced cancer‐specific survival, and CCL20‐mediated cytokine activation, highlighting its potential as a prognostic biomarker. Hsieh et al. [237] identified *F. nucleatum* as a GC‐specific biomarker, with diagnostic potential when combined with *Clostridium* species, in the context of declining *H. pylori* abundance during carcinogenesis. Despite mounting evidence implicating *F. nucleatum* in GICs, its pathogenic roles in gastrointestinal malignancies other than CRC remain insufficiently characterized. Recent clinical observations regarding *F. nucleatum* and related mechanisms in non‐CRC GICs are summarized in Table 2.

**TABLE 2:** A summary of clinical studies on *F. nucleatum* in GICs except CRC.

Cancer type	Author/year	Subjects	Region/country	Methods	Samples	Detection rate	Clinicopathological and molecular associations with *F. nucleatum*	References
EC	Ymamura et al. 2016	325 patients undergoing resection	Kumamoto, Japan	qPCR	Tumor tissues and AN tissues	23% (74/325)	*F. nucleatum* DNA enriched in EC; correlated with advanced stage and poorer survival through CCL20	[236]
	Ymamura et al. 2019	551 cases (207 in training cohort and 344 in validation cohort)	Japan	qPCR	Tumor tissues and AN tissues	−	Higher in EC patients; predicted poor recurrence free survival and chemoresistance in EC	[238]
	Nomoto et al. 2022	376 patients	Kumamoto, Japan	FISH and qPCR	FFPE tumor tissues and AN tissues	−	*F. nucleatum* DNA enriched in tumor; correlated with tumor depth and lymph metastasis via NOD1/RIPK2–NF‐κB pathway	[239]
	Wei et al. 2022	178 patients with EC and 101 healthy subjects	Guangdong, China	16S rRNA sequencing	Saliva	−	Higher *F. nucleatum* in saliva samples than that in healthy subjects; salivary *F. nucleatum* with two other bacterium could predict ESCC, with combined sensitivity 86.4%.	[240]
	Li et al. 2023	98 EC patients who received immunotherapy and 31 EC patients	Guangdong, China	ELISA, qPCR	Serum, tumor tissues and AN tissues	−	Higher *F. nucleatum* DNA and antibodies in nonresponders to PD‐1 inhibitors; its infection inversely affected αPD‐L1 efficacy in animals.	[143]
	Guo et al. 2023	22 patients	Guangdong, China	qPCR	Tumor tissues and AN tissues	−	Higher *F. nucleatum*‐DNA in tumors; positively correlated with METTL3 expression	[241]
	Zhang et al. 2023	107 patients	Hubei, China	FISH	FFPE cancerous and AN tissues	−	Clustered *F. nucleatum* enriched in tumor, correlating with deeper invasion, lymph metastasis, and advanced TNM stages; amplified the senescence‐associated secretory phenotype and increased DNA damage	[140]
	Kosumi et al. 2023	300 patients	Kumamoto, Japan	qPCR	FFPE tumor tissues	23% (70/300)	High *F. nucleatum* level correlated with reduced peritumoral lymphocytic reactions	[242]
	Liu et al. 2021	120 patients	Kumamoto, Japan	qPCR	FFPE tumor tissues	29% (35/120)	High *F. nucleatum* level correlated with poor chemotherapy response in ESCC by upregulating ATG7‐mediated autophagy to induce chemoresistance.	[136]
	Liang et al. 2022	258 patients	Henan, China	RNAscope	Paraffin‐embedded tumor tissues and AN tissues	32.95% (85/258) of tumor tissues and 3.49% (9/258) of paracancerous tissues	Association with high NLRP3 expression, myeloid‐derived suppressor cells enrichment, and shortened survival time	[124]
	Wang et al. 2022	196 patients	Henan, China	RNAscope	Paraffin‐embedded tumor tissues and AN tissues	32.29% (62/192) of tumor tissues and 4.17% (8/192) of paracancerous tissues	Association with poor prognosis, cisplatin resistance, and male smokers/drinkers by upregulating KIR2DL1 on CD8^+^ T cells.	[129]
	Li et al. 2021	111 patients	Henan, China	FISH and qPCR	Tumor and paired nontumor tissue samples	69.4% (68/98) of tumor tissues	*F. nucleatum* enrichment correlated with advanced pT stage, clinical stages, unique mutations, higher tumor mutation burden, and metastasis potential.	[243]
	Yin et al. 2023	65 patients	Hubei, China	FISH and qPCR	Paraffin‐embedded tumor tissues and AN tissues	−	Enriched in tumor and correlated with advanced stages, deeper tissue invasion, and promoted progression via AHR/CYP1A1/AKT signaling	[244]
	Lei et al. 2023	191 participants (109 early‐stage EC, 72 advanced EC, and 10 healthy control)	Chongqing, China	16S rRNA sequencing, FISH and qPCR	Paraffin tumor tissues and normal tissues	−	Higher abundance and detection frequency of *F. nucleatum* in advanced EC, correlating with tumor stage and driving EC via IL‐32/PRTN3–PI3K/AKT pathway	[245]
	Ding et al. 2023	73 patients	Shanxi, China	qPCR	Tumor tissues and AN tissues	−	Enriched in tumors, correlating with poor survival, and promoted proliferation via putrescine‐driven polyamine metabolism dysregulation	[117]
	Baba et al. 2023	306 patients	Kumamoto, Japan	qPCR	FFPE tumor tissues	21.2% (65/306) of tumors	Association with advanced stage, poor prognosis, and LINE‐1 hypomethylation, impacting prognosis but not affecting MGMT/MLH1 promoter methylation	[246]
	Nakajima et al. 2025	59 patients	Fukushima, Japan	qPCR and RNA‐ISH	Tumor tissues and AN tissues	59.3% (35/59) of tumors	Activated STING pathway, correlating with advanced stage and poor prognosis	[247]
	Sasaki‐Higashimoto et al. 2025	70 patients	Sendai, Japan	FISH and qPCR	FFPE tumor tissues, AN tissues, and metastatic lymph node	100% (70/70) of tumors in FISH; 68.6% (48/70) of tumors in qPCR	Associated with advanced stage, lymph node metastasis, and heterogeneous distribution patterns	[248]
GC	Coker et al. 2018	81 patients with superficial gastritis, AG, IM, and GC, Validation in 126 cases.	Xi'an and inner Mongolia, China	16S rRNA sequencing	Gastric mucosal samples	−	Enriched in the GC. Strong co‐occurrence network with disease progression	[249]
	Boehm et al. 2020	81 GC patients and 18 patients with normal gastric mucosa, 17 patients with chronic non‐AG and nine patients with AG/IM	Kaunas, Lithuania and Magdeburg, Germany	qRT‐PCR	Gastric biopsy specimens and paired tumor and AN tissues.	23.08% (18/78) of AN and 28.75% (23/80) of tumor samples 16.7% (3/18) of normal mucosa, 17.65% (3/17) chronic non‐AG mucosa and 0% (0/9) in AG/IM	Higher frequency and bacterial load in CRC than in GC patients; older age and worse overall survival in patients with Lauren's diffuse type; lower global long interspersed element‐1 DNA methylation	[84]
	Lehr et al. 2023	64 GC patients; validation in a cohort of 107 patients	Kaunas, Lithuania	16S rRNA sequencing	Paired gastric tissue and adjacent tissue samples	−	Higher abundance in GC patients	[250]
	Yamamura et al. 2017	20 GC patients	Kumamoto, Japan	qRT‐PCR	FFPE tumor tissue samples and matched normal FFPE specimens	10% (2/20) of tumor tissues and 0 of normal tissues	Higher level in superficial areas compared with the invasive areas	[251]
	Hsieh et al. 2018	Nine patients with gastritis, seven with IM and 11 GC patients	Taiwan	16S rRNA sequencing	Gastric biopsies	66.7% (6/9) of gastritis, 57.1% (4/7) of IM and 72.7% (8/11) of GC	Enriched in GC and with other bacteria as biomarker for GC	[237]
	Hsieh et al. 2022	36 GC patients	Taiwan	Nested PCR	Resected GC samples	44.4% (16/36)	Higher tumor mutation burden and poorer prognosis. Activation of ERBB2–PIK3–AKT–mTOR pathway	[252]
	Rodriguez et al. 2020	85 stomach adenocarcinoma cases in TCGA; validation in a cohort of 21 GC patients	Hawaii, USA	qRT‐PCR	FFPE tissue and TCGA cancer database	9% (8/85) cases in TCGA stomach adenocarcinoma; 14% (3/21) of GC in RTR cohort	−	[253]
	Liu et al. 2023	304 patients diagnosed with GC and developed venous thromboembolism complications	Henan, China	FISH	FFPE cancer tissues	34.5% (105/304)	Poorer prognoses and a disease course more frequently complicated by deep venous thromboembolism and pulmonary embolism	[254]
	Chen et al. 2022	120 patients with GC, 31 patients with AG, 35 patients with non‐AG, 26 patients with gastric polyps and 20 normal controls	Shandong, China	ddPCR, experiments in vitro	Saliva specimens	−	Association with TNM stage; promotion GC metastasis by accelerating the EMT process	[51]
	Hsieh et al. 2021	60 GC patients undergoing gastrectomy	Taiwan	Nested PCR	Resected frozen cancer tissues	31.7% (19/60)	Higher prevalence in GC and worse survival in GC patients cocolonized with *H. pylori*	[252]
	Hara et al. 2024	240 GC patients in cohort A and 115 GC patients in cohort B	Japan and USA	qRT‐PCR	FFPE and frozen biopsy	5.0% (12/240) in cohort A and 11.3% (13/115) in cohort B	Higher *F. nucleatum* DNA levels in tumor tissues than in the AN gastric tissues; association with poorer prognosis	[83]
	Zhang et al. 2025	40 patients in cohort 1 and 93 patients in cohort 2	Guangdong, China	16S rRNA sequencing, qRT‐PCR, and FISH,	Resected frozen cancer tissues and FFPE GC tissue microarray	−	*F. nucleatum* enrichment in metastatic GC correlated with advanced TNM staging, predicted postoperative recurrence, and reduced median survival and activates NF‐κB signaling to recruit protumoral neutrophils, driving PD‐L1‐mediated immune evasion.	[74]
Pancreatic cancer	Alkharaan et al. 2020	109 participants	Stockholm, Sweden	ELISA	Blood samples and saliva samples	−	Association with high‐risk intraductal papillary mucinous neoplasms tumors (high‐grade dysplasia/invasive cancer), evidenced by elevated plasma IgG and salivary IgA reactivity	[255]
	Hayashi et al. 2023	84 patients and 41 health controls	Fukuoka, Japan	qRT‐PCR	Tumors tissues and normal pancreatic tissues	15.5% (13/84) of pancreatic tumors and 7.3% (3/41) of normal	Correlated with larger tumors, retroperitoneal invasion, lymph node metastasis, and worse survival outcomes by inducing CXCL1 secretion and immunosuppressive microenvironment	[256]

*Abbreviations*: AG, atrophic gastritis; AN, adjacent normal; ddPCR, droplet digital PCR; EC, esophageal cancer; ELISA, enzyme‐linked immunosorbent assay; EMT, epithelial–mesenchymal transition; ESCC, esophageal squamous cell carcinoma; FFPE, formalin‐fixed paraffin‐embedded; FISH, fluorescence in situ hybridization; GC, gastric cancer; *H. pylori*, *Helicobacter pylori*; IM, intestinal metaplasia; qPCR, quantitative polymerase chain reaction; qRT‐PCR, quantitative reverse transcription PCR; TCGA, The Cancer Genome Atlas.

### 6.3 Head and Neck Cancers

Head and neck cancers (HNCs) ranks as the seventh most common cancer worldwide, affecting the oral cavity, oropharynx, nasopharynx, hypopharynx, larynx, nasal cavity, paranasal sinuses, and salivary glands. Approximately 90% of HNCs are squamous cell carcinomas, with oral squamous cell carcinoma (OSCC) being the most prevalent subgroup [257]. Extensive research has established microbial involvement in oropharyngeal carcinogenesis, with human papillomavirus (HPV) serving as a clinically validated etiological agent [258]. Chronic inflammation is recognized as a critical driver in carcinogenesis, with commensal microbiota hypothesized to bridge persistent inflammatory states and malignant transformation. Notably, periodontitis has been epidemiologically validated as an independent risk factor for oral cancer, and *F. nucleautm—*identified as a keystone pathogen in periodontitis—is therefore of particular interest in this context [259, 260, 261, 262, 263]. Though evidence regarding the association between elevated *F. nucleatum* abundance and clinical prognosis in HNCs remains mixed, with studies reporting positive correlations in some cohorts but no association in others, numerous studies have elucidated the association between *F. nucleatum* and HNCs and the mechanisms by which this anaerobe promotes tumor progression, as systematically summarized in Table 3.

**TABLE 3:** A summary of clinical studies investigating associations between *F. nucleatum* and HNCs.

Author/year	Number of patients	Country	Cancer type	Methods	Samples	Main reported findings	References
Chen et al. 2020	68	China	HNC	qPCR	Tumor tissues and the AN tissues	*F. nucleatum* enriched in tumors (more in nonsmokers), associated with lower stage, recurrence, better survival, and hypermethylation of tumor suppressor gene promoters	[113]
Neuzillet et al. 2021	212	France	HNC	qRT‐PCR	Tumor tissues	*F. nucleatum* correlated with lower alcohol intake, fewer nodal metastases, longer survival, less M2 macrophages	[264]
Desai et al. 2022	100	India	HNC	qRT‐PCR	Tumor tissues and AN tissues	*F. nucleatum* enriched in tumors, occurred mutually exclusive to HPV, and correlated with overexpression of inflammatory miRNAs, elevated innate immune cell fraction; association with poor survival, nodal metastases, and extracapsular spread in tongue tumors	[265]
Hsueh et al. 2022	231	China	HNC	qPCR	Tumor, para‐tumor tissues and vocal cord polyp tissues	*Fusobacterium*, particularly *F. nucleatum*, contributed to the aggressiveness of HNC by altering DNA mismatch repair and causing microsatellite instability, leading to poor prognosis in patients	[266]
Lau et al. 2023	82	China	Hypopharyngeal carcinoma	FISH	Tumor tissues and AN tissues	*F. nucleatum* enriched in tumor tissues and associated with worse prognosis and promoted oxidative stress via the miR‐361‐3p/NUDT1 axis.	[267]
Nie et al. 2024	10	China	OSCC	FISH	Tumor tissues and AN tissues	*F. nucleatum* enriched in OSCC, contributed to OSCC progression by promoting tumor cell proliferation, macrophage recruitment, and M2 polarization in vivo and in vitro	[120]
Guo et al. 2024	103	China	Nasopharyngeal carcinoma	FISH and 16S rRNA sequencing	Tumor tissues and AN tissues	*F. nucleatum* was prevalent in tumor tissues and promoted tumor progression and radioresistance through the SLC7A5/leucine–mTORC1 axis.	[268]
Ren et al. 2024	41	China	Laryngeal cancer	qRT‐PCR	Tumor tissues	*F. nucleatum* was detected in 41.5% (17/41) tumor samples and promoted the carcinogenesis through upregulating YWHAZ.	[269]
Datorre et al. 2025	94	Brazil	HNC	ddPCR	Tumor tissues	*F. nucleatum* presence was associated with a better prognosis. No significant association with clinicopathological features, TERTp, or HPV status.	[270]
Li et al. 2025	101	China	HNC	FISH	Tumour tissues and normal oral mucosa tissues	Elevated level of *F. nucleatum* in tumor increased the risk of tumor size, TNM stage, and metastasis. The abundance of *F. nucleatum* was found to predict worse survival rate and immunotherapy outcomes by promoting immunosuppressive phenotypes.	[146]
Muñoz‐Grez et al. 2025	31 (16 controls and 15 HNC patients)	Chile	OSCC	Computational proteomics	Tumour tissues and normal oral mucosa tissues	*F. nucleatum* dominated OSCC secretomes and drove OSCC progression via SLC7A11‐mediated glutamate efflux, fueling bacterial growth.	[271]

*Abbreviations*: AN, adjacent normal; FISH, fluorescence in situ hybridization; HNCs, head and neck cancers; HPV, human papillomavirus; OSCC, oral squamous cell carcinoma; qPCR, quantitative polymerase chain reaction; qRT‐PCR, quantitative reverse transcription PCR.

### 6.4 Other Cancers

Emerging research has linked *F. nucleatum* to a range of genitourinary malignancies, particularly in gynecological cancers such as ovarian cancer (OC), endometrial cancer, and cervical cancer (CC), with enrichment patterns varying across histological subtypes and tumor grades. In OC, nonserous histological subtypes exhibit higher *F. nucleatum* abundance compared with serous OC [272] and some cases of OC complicating *F. nucleatum* bacteremia have been documented [273, 274, 275]. In endometrial cancer, increased *F. nucleatum* levels in the cervicovaginal microbiota—especially in high‐grade tumors—suggest a potential causative or contributory role in tumorigenesis [276, 277]. In CC, high intratumoral *F. nucleatum* burden has been associated with relapse propensity, acquisition of cancer stem cell traits, and reduced survival, supporting its value as a potential diagnostic and prognostic biomarker [278]. Mechanistically, *F. nucleatum* can exacerbate lymph node metastasis in CC by activating TLR4/MAPK signaling via LPS, leading to upregulation of prometastatic factors such as EFNA1 and EDN2 in lymphatic endothelial cells [279], and may further promote HPV infection, persistence, and neoplastic progression by fostering a proinflammatory microenvironment and driving cancer‐associated metabolic reprogramming [280]. In addition, Rustetska et al. [281] found that *F. nucleatum* with *Pseudomonas aeruginosa* (*P. aeruginosa*) was identified as a tumor‐promoting bacterium in vulvar squamous cell carcinoma, correlating with shorter time to disease progression and poor patient survival. Its presence associated with increased CD66b^+^ neutrophil infiltration and elevated expression of neutrophil serine proteases within tumor microabscesses—features linked to adverse outcomes. In male patients, *F. nucleatum* is significantly enriched in prostate tissues affected by benign prostatic hyperplasia, inflammation, and cancer, indicating a potential role in prostatic disease pathogenesis, although no significant differences in abundance have been reported across disease states [282].

In other female malignancies, *F. nucleatum* has been linked to BC. Initial observations link periodontitis‐associated *F. nucleatum* in subgingival biofilms to elevated BC risk, a relationship supported by subsequent detection of this bacterium within breast tumor tissues [283, 284]. In vivo and in vitro studies reveal that the surface adhesin Fap2 of *F. nucleatum* binds to elevated polysaccharide d‐galactose‐β (1‐3)‐N‐acetyl‐D‐galactosamine (Gal‐GalNAc) in BC tissues, enabling colonization and accelerating tumor progression, while *F. nucleatum*‐derived small EVs promote oncogenic behaviors in BC cells via TLR4‐dependent signaling [285, 286]. Additionally, *F. nucleatum* facilitates BC immune evasion by suppressing CD8^+^ T cell cytotoxicity via the NF‐κB/PD‐L1 pathway [142]. Multiomics analysis shows that in BC, *F. nucleatum* localizes preferentially to tumor cell‐rich regions, where it significantly modulates the expression of RNAs and proteins involved in proliferation, migration, and invasion, notably upregulating MAPK pathway components, VEGFD, and PAK1 [287].

## 7 *F. nucleatum* in Other Systemic Diseases and Conditions

### 7.1 Adverse Pregnancy Outcomes

*F. nucleatum*, the most prevalent oral pathogen linked to APOs, is frequently detected in placental/fetal tissues—including amniotic fluid, fetal membranes, cord blood, neonatal gastric aspirates—and has been associated with a spectrum of APOs, such as preterm birth, preterm premature rupture of membranes, chorioamnionitis, stillbirth, preeclampsia, early‐onset neonatal sepsis, and intrauterine growth restriction, either as a sole pathogen or within polymicrobial infections [288].

Multiple studies have demonstrated that *F. nucleatum* enrichment in the oral and vaginal microbiota of pregnant women correlated with an increased risk of preterm birth, suggesting its potential utility as a predictive biomarker for preterm delivery and neonates health [289, 290, 291, 292]. Emerging evidence shows that *F. nucleatum* detected in the neonatal microbiome may originate from maternal oral cavity via hematogenous dissemination, rather than urogenital tract and intestine—a hypothesis validated in animal models [293, 294]. In murine experiments, intravenous injection of either *F. nucleatum* alone or subgingival plaque samples containing *F. nucleatum* into mice resulted in placental colonization [295, 296]. This colonization may be mediated by bacterial surface adhesins FadA, Fap2, and RadD [297, 298, 299]. Recent work proves that environmental ethanolamine‐dependent formation of bacterial microcompartments is crucial for *F. nucleatum* pathogenicity in a mouse model of preterm birth [300].

TLRs, key pattern recognition receptors on mammalian cell surfaces, are central to host inflammatory responses. Multiple studies have implicated TLRs, particularly TLR2 and TLR4, in mediating *F. nucleatum*‐induced proinflammatory responses in diverse inflammatory diseases [186, 301, 302, 303, 304, 305, 306]. However, TLR4‐mediated inflammation triggered by *F. nucleatum* does not directly account for fetal death in APO models [307]. In contrast, TLR2/4 activation can induce regulatory T cells that suppress intestinal inflammation caused by *F. nucleatum* [206].

### 7.2 Alzheimer's Disease and Neuroinflammation

Alzheimer's disease (AD), the most common form of dementia in older adults, is characterized by progressive cognitive decline, with 95% of cases being late‐onset sporadic forms driven by aging, genetic susceptibility, and modifiable risk factors [308]. Accumulating evidence links periodontal pathogens to AD pathogenesis, with *F. nucleatum* emerging as a notable contributor. Elevated antibody levels against *F. nucleatum* and oral *F. nucleatum* load levels have been reported in AD patients compared with cognitively healthy controls [309, 310, 311]. In vivo, *F. nucleatum* activated microglial cells‌, causing morphological changes, accelerating proliferation, and enhancing the expression of TNF‐α and IL‐1β. In mice models, *F. nucleatum*‐induced periodontitis exacerbated AD‐like symptoms‌, including cognitive impairment, β‐amyloid accumulation, and Tau protein phosphorylation, which is wildly believed to be associated with the local and surrounding inflammatory environment, especially in response to Gram‐negative bacterial stimuli [312]. In rats model, *F. nucleatum* impaired cognitive performance and promoted Alzheimer's‐like pathology by elevating ‌Aβ1‐42‌ and ‌p‐Tau181‌ expression. Notably, although the bacterium does not directly invade the brain, it provokes systemic effects such as increased serum LPS levels and gut microbiota dysbiosis—characterized by enrichment of *Streptococcus* and *Prevotella*, taxa associated with neurotoxin production. In parallel, *F. nucleatum* perturbs host metabolic pathways such as ‌amino acid degradation‌ and ‌carbohydrate metabolism‌, potentially aggravating metabolic disorders linked to neurodegeneration [313]. Beyond the bacterium itself, circulating bacterial toxins and OMVs may also amplify central nervous system damage by sustaining inflammatory cascades [314, 315]. The precise contribution of *F. nucleatum* virulence factors to AD progression remains to be fully elucidated.

Preclinical evidence also supports a direct role for *F. nucleatum* in neuroinflammation through bacterial translocation. In a rat model combining induced periodontitis and chronic stress, *F. nucleatum* was uniquely detected in brain tissue, suggesting hematogenous spread via a “leaky mouth” mechanism. This group displayed the most severe neuroinflammatory profile, with markedly elevated proinflammatory mediators, increased microglial activation, and pronounced blood–brain barrier disruption in the frontal cortex. Mechanistically, the translocation of *F. nucleatum* coincides with dysregulated tight junction proteins, increased adhesion molecules, and MMP9 overexpression. These alterations facilitate bacterial entry and amplifies hypothalamic‐pituitary‐adrenal axis hyperactivity. The spatial and biochemical association between *F. nucleatum* presence, blood–brain barrier breakdown, and neuroinflammation positions this pathogen as a potential biological bridge linking periodontal infection to depression‐associated central nervous system pathology [316, 317].

### 7.3 Metabolic and Liver Diseases

*F. nucleatum* is reported to serve as a contributor to systemic metabolic dysregulation and liver pathology, acting primarily through its involvement in periodontal disease and disruption of the gut–liver axis. In patients with metabolic syndrome, *F. nucleatum* is the most prevalent periodontal pathogen detected in gingival crevicular fluid, with abundance positively correlated with elevated inflammation markers, increased fat mass, and visceral adiposity. Lifestyle interventions such as anti‐inflammatory diets and regular exercise have been shown to reduce *F. nucleatum* levels in parallel with improvements in metabolic syndrome parameters [318]. Mechanistically, *F. nucleatum* translocates from the oral cavity to the liver, where it activates the PI3K/Akt/mTOR signaling pathway in hepatocytes, driving glycolysis and de novo lipogenesis—resulting in hypertriglyceridemia, hepatic cholesterol accumulation, and exacerbated atherosclerosis in murine models [188]. In acute liver failure, *F. nucleatum* exacerbates hepatic inflammation and disrupts energy homeostasis by inhibiting the NAD^+^ salvage pathway, thereby reducing NAD^+^ levels and suppressing the SIRT1/AMPK signaling. This metabolic blockade accelerates hepatocellular injury and is accompanied by enhanced macrophage infiltration and proinflammatory cytokine release, further driving disease progression [319].

### 7.4 Respiratory Diseases

*F. nucleatum* also has been associated with several major respiratory diseases, including pneumonia, chronic obstructive pulmonary disease (COPD), and lung cancer. It is frequently detected in respiratory samples such as tracheal aspirates, bronchoalveolar lavage fluid, and pleural effusions from affected patients. Clinical observations indicate that its presence correlates with poorer outcomes, including decreased lung function during COPD exacerbations, diminished responsiveness to immunotherapy in lung cancer, and poorer antibiotic treatment efficacy. Mechanistically, *F. nucleatum* contributes to respiratory pathology by inducing mucus hypersecretion and sustaining chronic airway inflammation through upregulation of proinflammatory cytokines. In addition, it exhibits synergistic interactions with other respiratory pathogens—most notably *P. aeruginosa—*to enhance biofilm formation and amplify virulence [69].

### 7.5 Irritable Bowel Syndrome

Irritable bowel syndrome (IBS) is a common functional gastrointestinal disorder characterized by recurrent abdominal pain and altered bowel habits. Experimental evidence shows that oral administration of *F. nucleatum* significantly worsens visceral hypersensitivity in maternally separated rats—an established IBS model—while altering gut microbiota diversity and composition, despite the bacterium itself not colonizing the gut. Notably, *F. nucleatum* elicits the production of specific secretory IgA antibodies in both rat models and IBS patients, predominantly directed against its outer membrane protein FomA. The immune response, rather than direct bacterial colonization, appears to underlie its pathogenic role and correlates positively with symptom severity and psychological scores [320].

## 8 Mechanisms of Host–Pathogen and Microbe–Microbe Interactions

### 8.1 Host–Pathogen Interactions

Recent studies have expanded our understanding of the mechanisms underlying *F. nucleatum* interaction with host (Figure 3B). Beyond the extensively characterized FadA–E‐cadherin interaction, emerging evidence suggests that *F. nucleatum* engages with host cells through multiple additional adhesins and virulence determinants. Abed et al. [321] demonstrated that the tumor‐specific colonization could be mediated through *F. nucleatum* recognition and attachment to the host Gal‐GalNAc, which is overexpressed in CRC, GC, and BC tissues by *F. nucleatum* displayed Fap2 [285, 322]. Notably, Gal‐GalNAc levels are substantially higher in breast and colon adenocarcinomas compared with their respective normal tissues, whereas in gastric tissues, Gal‐GalNAc abundance is already elevated in normal controls and remains comparable to that in gastric adenocarcinomas—potentially explaining tumor‐type differences in *F. nucleatum* load [322]. Moreover, certain host genetic mutations can enhance susceptibility to Fap2‐mediated colonization [323]. We summarize the known virulence proteins of *F. nucleatum* and their corresponding host receptors, along with the implications of these interactions in Table 4.

**TABLE 4:** A summary of surface proteins of *F. nucleatum*, their corresponding host receptors, and functional implications.

Virulence proteins	Host receptors	Implications	References
FadA	VE‐cadherin	Mediates adherence to/invasion of endothelial cells; increases endothelial permeability	[87]
	E‐cadherin	Adheres to/invades CRC cells; upregulates Annexin A1; activates β‐catenin signaling; promotes carcinogenesis and cancer progression	[6, 97]
Fap2	Gal‐GalNAc	Mediates colonization of tumors and the placenta; promotes cancer cell migration	[79, 285, 321]
	TIGIT	Inhibits NK and T cell cytotoxicity, promoting tumor immune evasion	[125]
RadD	CD147	Facilitates the attachment to CRC cells; activates PI3K–AKT–NF‐κB–MMP9 cascade enhancing tumorigenesis	[80]
	Siglec‐7	Inhibits NK cell‐mediated cancer cell killing	[126]
Gbp	CypA	Activates PI3K–AKT/MAPK/NF‐κB pathways promoting inflammation and lipid deposition	[4]
	Annexin A2	Inactivates Wnt/β‐catenin pathway inhibiting osteogenic differentiation	[174]
CbpF	CEACAM1	Inhibit immune cell activity	[127, 324, 325, 326]
FomA (via EVs)	TLR2	Triggers innate immunity by promoting NF‐κB activation via the dynamin‐mediated endocytosis	[327]
FN1441	FomA (transferred by EVs)	Facilitates *F. nucleatum* autoaggregation; mediates bacterial adhesion and colonization	[328]

*Abbreviations*: CEACAM1, carcinoembryonic antigen related cell adhesion molecule 1; CRC, colorectal cancer; EVs, extracellular vesicles; Gal‐GalNAc, D‐galactose‐β(1‐3)‐N‐acetyl‐D‐galactosamine; NK, natural killer; TIGIT, T cell immunoglobulin and ITIM domain; TLR2, toll‐like receptor 2; VE‐cadherin, vascular E‐cadherin.

In addition to virulence factors with well‐defined host receptors, *F. nucleatum* also produces proteins that exert potent pathogenic effects despite their receptors remaining unidentified. Virulence‐associated proteins such as Dps and GroEL also contribute to host interactions. Dps, identified from the culture supernatant, lyses and disrupts erythrocytes by the competition for iron acquisition and facilitates intracellular survival in macrophages by upregulating the expression of the chemokine CCL2/CCL7, thereby promoting CRC metastasis through EMT [329]. GroEL, the heat‐shock protein of *F. nucleatum*, contributes to atherosclerosis progression through molecular mimicry‐induced autoimmunity and direct activation of proinflammatory and prothrombotic pathways in endothelial cells, further coupled with foam cell formation [187].

Beyond direct cellular adhesion and invasion, *F. nucleatum* further through its secreted metabolites and EVs modulates host inflammatory responses and oncogenesis as we described above.

### 8.2 Microbe–Microbe Interactions

Beyond its role in biofilm formation via physical coaggregation and metabolic cross‐feeding with commensal microbiota under physiological conditions, *F. nucleatum* further engages in interactions with local microbial communities to underpin disease progression in pathological states (Figure 3B).

#### 8.2.1 Oropharyngeal Bacteria

*F. nucleatum*, a core oral microbiota component, not only exerts direct pathogenicity but also serves as a physical and metabolic bridge between microbial colonizers, enabling synergistic virulence through interspecies interactions. A well‐studied example is its partnership with *P. gingivalis*, a keystone Gram‐negative anaerobe in periodontitis and a member of the “red complex.” *P. gingivalis* enhances *F. nucleatum* growth, while *F. nucleatum* sustains *P. gingivalis* viability in oxygenated environments [330, 331]. Their coinfection suppresses host immunity, boosts adhesion and invasion efficiency of *P. gingivalis*, and exacerbates alveolar bone loss and amplify proinflammatory cytokine production in vivo [172, 332, 333].

Beyond *P. gingivalis*, *F. nucleatum* facilitates the pathogenicity of otherwise less‐invasive species. For instance, it enables *Streptococcus cristatus* and *Streptococcus sanguinis* to adhere to and invade oral epithelial cells [334]. Coaggregation between *Fnp* and *Streptococcus gordonii* reduces the adhesion and invasion of *Fnp*’s own epithelial adhesion and invasion but enhances the adhesion of *Streptococcus gordonii*. Furthermore, this interaction synergistically promotes the secretion of TNF‐α and IL‐6 from gingival epithelium through TLR/NF‐κB and TLR/MAPK signaling while suppressing the release of transforming growth factor‐β1, amplifying inflammation. Coaggregation also reprograms bacterial transcriptional profiling, enhances intracellular survival in macrophages, and suppresses macrophage bactericidal and proinflammatory responses [335, 336]. A similar suppression pattern occurs with *P. gingivalis* coculture systems, which inhibits *F. nucleatum* invasion by downregulating its adhesions FadA and FomA via proteases in *P. gingivalis*‐derived OMVs, while *F. nucleatum* reciprocally promotes *P. gingivalis* invasion during coinfection [337].

Metabolic cross‐feeding further reinforces these partnerships. *F. nucleatum* acquires proteolytic capacity via *P. gingivalis*‐activated plasmin, enabling nutrient acquisition for the community [338], and converts amino acids from other commensals into polyamines that accelerate *P. gingivalis* biofilm maturation and dispersal, creating pathogenic microenvironment [339]. Additionally, *F. nucleatum* abundance in the oropharynx correlates positively with *Neisseria meningitidis* carriage, likely through propionic acid cross‐feeding that supports meningococcal growth [340].

#### 8.2.2 Gastrointestinal Bacteria

*H. pylori* is a well‐established risk factor for GC, yet its interplay with other gastric microorganisms, particularly *F. nucleatum*, remains unclear. Several studies have noted a decrease in the abundance of *H. pylori* in GC patients, accompanied by increased *F. nucleatum* levels [251]. A microbial succession hypothesis has been proposed to explain the observed decline of *H. pylori* and the subsequent proliferation of nondominant flora in GC. *H. pylori* survives in the stomach by producing urease, which increases the periplasmic pH and triggers a strong inflammatory response that undermines the gastric mucosal barrier. This disruption facilitates the invasion of secondary bacteria into the mucus layer, potentially contributing to the malignant transformation. Evidence suggests possible sequential or synergistic roles for *H. pylori* and *F. nucleatum* in GC progression. *F. nucleatum* colonization has been linked to poorer prognosis in *H. pylori*‐positive patients with advanced‐stage GC [252, 341]. However, other data indicate a mutually exclusive pattern: in a cohort of GC and esophagogastric junction cancer cases with *H. pylori* infection in the normal gastric epithelium, *F. nucleatum* is rarely detected in cancerous regions [83]. Although *H. pylori* may provide a less acidic environment that theoretically facilitate *F. nucleatum* biofilm formation and enhance adhesion, the infection rate and bacterial loads of *F. nucleatum* do not appear to increase in an alkaline environment due to atrophic gastritis or intestinal metaplasia [342]. Further studies are required to clarify whether these organisms interact cooperatively, sequentially, or competitively in GC pathogenesis.

In the intestine, *F. nucleatum* also engages in pathogenic partnerships with other bacteria. *Clostridioides difficile*, a spore‐forming, anaerobe responsible for severe diarrhea. *F. nucleatum* can promote *Clostridioides difficile* colonization and biofilm formation in the intestinal mucus layer through RadD adhesin‐mediated coaggregation and flagella‐dependent interactions while enhancing extracellular polysaccharide production [343].

#### 8.2.3 Vaginal Microbiota

*Gardnerella vaginalis*, a key species associated with BV, may engage in symbiotic biofilm formation with *F. nucleatum*, potentially enabling them to outcompete protective lactobacilli within the vaginal niche [344]. Sialidases plays multifaceted roles in bacterial‐host interactions, coinfections, and dysbiosis within the oral cavity, GIT, and respiratory systems. Notably, detectable sialidase activity in vaginal secretions represents a biochemical hallmark of BV. While *F. nucleatum* itself does not produce sialidase, it frequently colonizes niches rich in sialidase‐producing bacteria, including the oral, gut, and vaginal environments. Agarwal et al. [70] revealed that the relationship between *F. nucleatum* and sialidase‐producing bacteria was mutualistic rather than unidirectional, involving metabolite cross‐feeding that reciprocally promoted growth and persistence. Within the vaginal microbiota, this interaction fosters a dysbiotic state characterized by elevated sialidase activity and increased *Gardnerella vaginalis* abundance, which in turn enhances the niche's susceptibility to *F. nucleatum* colonization. This positive feedback loop may contribute to BV pathogenesis and facilitate opportunistic infections.

#### 8.2.4 Other Microbe

*P. aeruginosa*, a prevalent pathogen in chronic respiratory infections, exhibits enhanced proliferation and structurally/functionally complex biofilm formation when cocultured with *F. nucleatum*. The coexistence of *F. nucleatum* and *P. aeruginosa* enhances the invasive ability of both bacteria and exacerbates lung damage, particularly in COPD patients. Additionally, *F. nucleatum* amplifies the proinflammatory cytokine secretion and cytotoxicity induced by *P. aeruginosa*, contributing to a rapid decline in lung function [69].

*Candida albicans*, an opportunistic pathogenic yeast commonly colonizing the GIT and oral cavity, contributes to carcinogenesis through alcohol dehydrogenase‐mediated acetaldehyde production. *F. nucleatum* coaggregates with *Candida albicans*, facilitating mucosal colonization and potentially heightening oral cancer risk by increasing host exposure to acetaldehyde [222].

## 9 Key Debates and Unresolved Questions

### 9.1 Causality versus Passenger/Enabler

Despite growing evidence linking *F. nucleatum* to CRC and various inflammatory diseases, a fundamental question persists: is *F. nucleatum* a direct causal driver of disease, or merely an opportunistic passenger/enabler exploiting a compromised microenvironment? This distinction is pivotal for elucidating pathogenesis and guiding targeted interventions.

In CRC, the argument for *F. nucleatum* as an active driver is strengthened by its specific association with oncogenic events. Critically, FadA gene levels increase stepwise from healthy colon tissue to adenoma to carcinoma, with even histologically “healthy” tissue adjacent to lesions showing elevated FadA compared with true healthy controls [345]. This mirrors findings that *F. nucleatum* is enriched in precancerous lesions and correlates with nuclear β‐catenin localization [99]. Mechanistically, *F. nucleatum* not only colonizes but actively manipulates host signaling, as FadA–E‐cadherin binding disrupts tumor suppression, activates β‐catenin signaling, and promotes Wnt/β‐catenin‐driven oncogene expression [346]. It has also been implicated in promoting CIMP, MSI, and BRAF/TP53 mutations [347]. Together, these observations support a model in which *F. nucleatum* functions as a second “hit”—with host genetic alterations creating susceptibility, and bacterial infection delivering a subsequent oncogenic trigger [97].

A similar driver‐like role may operate in inflammatory diseases, where *F. nucleatum* can actively induce excessive inflammation when epithelial integrity is compromised or microbial dysbiosis occurs, thus amplifying pathology rather than merely exploiting pre‐existing lesions.

Nonetheless, the passenger hypothesis cannot be dismissed. *F. nucleatum*’s enrichment in advanced tumors and its capacity to reshape the TME suggest it may preferentially expand after malignant transformation, acting as an enabler of progression, metastasis, and therapy resistance rather than an initiator. Longitudinal studies are urgently needed to clarify whether *F. nucleatum* colonization precedes driver mutations and early neoplastic events, or whether it predominantly follows them.

### 9.2 Origin of Extra‐Oral *F. nucleatum* and Strain Variation

The consistent enrichment of *F. nucleatum* in CRC tissues raises a critical question about its origin. Although *F. nucleatum* is a core oral commensal, compelling evidence suggests that CRC‐associated strains may originate from the oral cavity [86]. Oral‐derived *F. nucleatum* may disseminate via ingestion through the digestive tract, hematogenous spread through transient bacteremia, comigration with host cells, or—less commonly—direct mucocutaneous contact with infected oral sites. However, the primary transmission route remains unclear.

Emerging evidence reveals spatial and disease‐specific heterogeneity in *F. nucleatum* distribution across host niches, potentially attributable to divergent pathogenic capabilities among subspecies or strains. Historically, *F. nucleatum* was classified into subspecies, but genomic analyses now reveal substantial strain‐level variation within subspecies. In particular, *Fna* can be subdivided two clades: *Fna* C1 (primarily oral) and *Fna* C2 (enriched in CRC). *Fna* C2 predominates within CRC TME, harboring clade‐specific genetic features that enhance metabolic adaptability and virulence potential. This strain‐level variation provides a plausible explanation for inconsistencies in *F. nucleatum*‐associated phenotypes reported in earlier studies. It also positions *Fna* C2 as the primary pathogenic subgroup, underscoring its relevance as a mechanistic focus and therapeutic target in CRC research [14, 348].

### 9.3 Clinical Translation: Biomarker and Target Validation

*F. nucleatum*’s strong association with CRC progression positions it as a promising diagnostic biomarker and therapeutic target. Quantification of *F. nucleatum* in fecal samples has shown encouraging diagnostic performance. For example, Liang et al. [349] reported 77.7% sensitivity and 79.5% specificity for CRC detection, while Wong et al. [350] reported sensitivities of 73.1% for CRC and 15.5% high‐grade adenomas. However, detection rates differ across cohorts, reflecting population‐specific microbiome differences and heterogeneity in detection methodologies. These findings highlight the need for standardized, validated protocols and large‐scale, multiethnic studies before *F. nucleatum*‐based assays can be integrated into clinical CRC screening programs [223].

### 9.4 Methodological Limitations

Key methodological gaps impede definitive conclusions about *F. nucleatum*’s role in CRC, with detection inconsistencies representing a major challenge. The wide variability in *F. nucleatum* prevalence stems from heterogeneity in: sampling sources (tissue vs. stool vs. plasma), sampling processing (formalin‐fixed paraffin‐embedded vs. fresh‐frozen samples), and detection methods (PCR vs. fluorescence in situ hybridization vs. next‐generation sequencing). Moreover, new studies emphasize spatial heterogeneity in *F. nucleatum* distribution within tumors and functional variation among bacterial subpopulations, suggesting that single‐site or single‐method detection may underestimate its true prevalence and biological impact. Standardization of sampling protocols, processing techniques, and analytical pipelines is therefore essential to ensure cross‐study comparability and accurate interpretation [225, 348].

## 10 Therapeutic and Preventive Strategies Targeting *F. nucleatum*

Although *F. nucleatum* exists as a commensal organism under physiological conditions, its strong association with tumor progression and poor prognosis across multiple malignancies has raised considerable concern. Targeting and eradicating *F. nucleatum* therefore presents a promising avenue for cancer therapy. Several studies have shown that interventions against *F. nucleatum* infection can inhibit tumor development and improve patients outcomes. Below, we summarize current and emerging strategies targeting *F. nucleatum*.

### 10.1 Antimicrobial Approaches

Antibiotics remain the primary therapeutic option against bacterial infection. As an anaerobic bacterium, *F. nucleatum* is highly sensitive to nitronidazoles. In murine models, metronidazole administration significantly reduced the intratumoral *F. nucleatum* load, inhibited the tumor growth, and mitigated *F. nucleatum*‐induced resistance to chemotherapy and immunotherapy [136]. To enhance antibacterial efficacy, novel delivery systems—such as liposomal, gel, and phage—have been developed to improve sustained drug release, bioavailability, and half‐life [351, 352, 353]. The pH‐responsive nanoassembly can selectively disassemble in the acidic TME, releasing antibacterial agents that eradicate intratumoral *F. nucleatum* [354]. In addition, an antibacterial nanoplatform utilizing ultrasound‐generated ROS to eliminate intratumoral *F. nucleatum*, augments sonodynamic therapy efficacy against CRC, inhibits metastasis, and reduces skin phototoxicity [355].

However, the timing of antibiotic administration appears critical. Lessons from *H. pylori* management in GC can provide a valuable precedent: eradication therapy postgastrectomy improves survival, and *H. pylori* status serves as a prognostic indicator after curative resection [356, 357]. In the case of *F. nucleatum*, metronidazole confers protective effects only when administered prior to tumor resection, whereas postsurgical treatment does not yield comparable benefits—a difference potentially attributable to variations in tumor burden and immune status [351]. These comparisons underscore the importance of optimizing therapeutic timing to maximize bacterial eradication and improve clinical outcomes.

### 10.2 Targeting Virulence Factors

The pathogenicity of *F. nucleatum* is largely driven by LPS and its virulence proteins. Antimicrobial peptides like Br‐J‐I can disrupt *F. nucleatum* membranes by direct interaction with these virulence factors, synergistically enhancing the efficacy of chemotherapy [358]. Blocking key adhesins offers another promising strategy. Competitive inhibition of adhesion molecules can prevent *F. nucleatum* from binding to host cells, thereby limiting bacterial invasion and dampening inflammatory responses. For instance, specific free glycans terminating in galactose or N‐acetylgalactosamine can bind to the Fap2 adhesin on *F. nucleatum*, competitively blocking its interaction with host glycans carrying the same terminal motifs [95]. Additionally, targeting essential bacterial enzymes required for survival and virulence has been proposed as a means to selectively impair *F. nucleatum* viability [231].

### 10.3 Microbiome Modulation

Microbiome‐based strategies aim to suppress *F. nucleatum* pathogenicity by restoring a balanced microbial community [359]. Probiotics inhibit pathogens through the production of antibacterial compounds, modulation of host immune responses, direct antagonistic interactions, and competitive exclusion. For example, *Bifidobacterium animalis*, as a potential probiotic, inhibits *F. nucleatum* growth in coculture by competing for nutrients and producing acidic metabolites from amino acid and carbohydrate metabolism, creating an unfavorable environment for the pathogen—although metabolic cross‐feeding indicates potential bidirectional interactions [360]*. Akkermansia muciniphila*, an intestinal probiotic constituting 3% of healthy human colonic microbiota, suppresses *F. nucleatum* growth and virulence gene expression while concurrently attenuating the *F. nucleatum*‐induced TLR4/MyD88/NF‐κB activation in gingival epithelial cells, thereby reducing periodontal bone loss and soft tissue inflammation in vivo [301]. Zhu et al. [361] demonstrated that hyaluronic acid‐inulin coated *Enterococcus faecium* could target and suppress *F. nucleatum* by specifically adhering to colon tumor tissues and inhibiting *F. nucleatum* proliferation. Additionally, *Saccharomyces cerevisiae* JKSP39, a potential probiotic yeast strain ameliorates *F. nucleatum*‐associated colitis by enhancing antioxidant defenses, restoring gut barrier integrity, and modulating pro‐/anti‐inflammatory cytokine balance [362]. Beyond probiotics, restoring microbiota composition through fecal microbiota transplantation has shown potential—clearing *F. nucleatum* colonization in pediatric patients when donors are *F. nucleatum*‐negative [363].

Prebiotics and postbiotics may both play role in combating *F. nucleatum*. Agarooligosaccharides, a novel prebiotic, selectively inhibits *F. nucleatum* by downregulating fatty acid biosynthesis genes, altering membrane composition, and suppressing growth [364]. Butyrate, a key postbiotic SCFA derived from dietary fiber fermentation, suppresses adhesion‐associated outer membrane proteins in *F. nucleatum*, thereby reducing growth, colonization in colorectal tissues, and *F. nucleatum*‐induced chemoresistance [365].

### 10.4 Vaccines

Vaccine strategies against *F. nucleatum* primarily focus on key virulence factors such as FomA and Fap2, or employ whole inactivated bacteria to stimulate protective immunity. These vaccines, delivered via recombinant probiotics (*Lactobacillus*), nanoparticles, or fusion proteins, have shown the ability to induce robust antibody production and T cell activation, thereby reducing bacterial colonization, interspecies coaggregation, and abscess formation in preclinical models [366, 367, 368, 369, 370, 371, 372, 373]. While these findings are encouraging, challenges remain in optimizing delivery systems, minimizing off‐target effects, and establishing efficacy and safety in clinical settings.

### 10.5 Adjunctive Therapy in Cancer

*F. nucleatum*’s established role in tumor progression makes it a high‐value target for adjunctive cancer therapy. The organic polymers exert dual antibacterial and antitumor effects by facilitating tumor cells endocytosis, preventing *F. nucleatum*‐promoted autophagy, and increasing intracellular ROS [354, 374]. Liposome‐based antibiotic delivery not only eliminates *F. nucleatum* but also releases tumor neoantigens, activating CD8^+^ T cells against both infected and uninfected tumor cells [351]. Phages and the antimicrobial peptides, when combined with chemotherapy or PD‐1 inhibitors, can selectively eliminate intratumoral *F. nucleatum* and enhance cancer therapeutic efficacy [353, 358]. Innovative *F. nucleatum‐*mimicking nanomedicine, created by fusing bacterial membranes with colistin‐loaded liposomes, offers targeted bacterial clearance while preserving gut microbiota and improving immunotherapy response [375]. An optogenetic system drives cancer‐associated fibroblasts within the tumor to locally produce the human antibacterial peptide LL37. This LL37 depletes intratumoral *F. nucleatum* while preserving the microbiota due to its localized production and intercellular trafficking, thereby promoting prognosis of cancer patients [376].

### 10.6 Preventive Measures

Preventive strategies aim to limit *F. nucleatum* reservoirs and restrict its systemic dissemination. Maintaining good oral hygiene can reduce periodontal niches, lowering the risk of bacterial translocation to extraoral sites [3]. As proinflammatory diets significantly increase the risk of *F. nucleatum*‐positive colorectal tumors, adopting anti‐inflammatory diets may reduce *F. nucleatum* colonization by lowering systemic inflammation and preserving gut barrier integrity, thereby preventing *F. nucleatum*‐driven carcinogenesis [377]. In addition to these lifestyle measures, innovative localized antimicrobial approaches offer promising preventive potential. For example, 5‐aminolevulinic acid can be converted by *F. nucleatum* into photosensitive porphyrins, which—upon light activation—generate ROS that selectively kill the bacterium. This targeted photodynamic effect reduces *F. nucleatum* abundance in the oral microbiota while preserving overall microbial diversity, thereby controlling the pathogen at its source without inducing broad‐spectrum microbial disruption. Such oral‐site interventions may help interrupt *F. nucleatum*’s dissemination to extra‐oral sites and reduce its contribution to both nonmalignant inflammatory conditions and cancer progression [378].

Overall, these strategies offer distinct advantages regarding specificity and drug delivery efficiency; however, more in vitro and in vivo validation is essential to confirm their efficacy, safety, and long‐term benefits.

## 11 Summary and Future Perspectives

This review consolidates current evidence positioning *F. nucleatum* as a commensal oral biofilm architect and an opportunistic pathogen with broad systemic relevance. Under healthy conditions, *F. nucleatum* functions as an oral commensal, primarily residing within oral biofilms to maintain homeostasis and rarely colonizing extra‐oral sites. In pathological contexts, however, *F. nucleatum* is implicated in a spectrum of nonmalignant inflammatory diseases and malignancies—most notably CRC—through mechanisms including modulation of host signaling pathways, promotion of metastasis, immune evasion, and induction of therapy resistance. Significant strain heterogeneity underlies these effects, with niche‐specific adaptations such as the CRC‐enriched *Fna* C2 clade. Therapeutic strategies under investigation range from antimicrobials and adhesin blockers to microbiota modulation, vaccination, and preventive measures, some of which also function as adjunctive cancer therapies.

Despite rapid progress, fundamental uncertainties remain. Foremost is the unresolved question of pathogenic causality—whether *F. nucleatum* acts as an active driver of tumor initiation, consistent with a “two‐hit” model in CRC, or as a passenger that opportunistically thrives in pre‐existing disease microenvironments. Clarifying this requires longitudinal, multiomics studies linking temporal colonization patterns to molecular tumorigenesis. Strain‐ and clade‐specific pathogenic mechanisms remain incompletely characterized; deeper molecular dissection of virulence determinants and their host interactions is critical. Moreover, the associations between *F. nucleatum* abundance, specific subspecies, and diverse disease phenotypes are confounded by host genetics, ethnicity, diet, medication use, and technical heterogeneity in microbiome profiling. Standardized methodologies for sampling, processing, and analysis are urgently needed to enable reproducible, cross‐cohort comparisons.

The strong association between *F. nucleatum* and CRC progression highlights it*s* promise as a biomarker for disease preventing, early detection, and prognostication—paralleling the clinical utility of *H. pylori* in GC. However, turning this potential into real‐world medical use faces several challenges: (i) lack of standardized, clinically validated detection platforms capable of distinguishing pathogenic from commensal strains; (ii) limited prospective data demonstrating predictive value across populations; and (iii) uncertainty regarding the most effective therapeutic window and delivery strategies for *F. nucleatum*‐targeted interventions. Addressing these gaps offers an opportunity to integrate microbial diagnostics into precision oncology, develop targeted microbial clearance approaches, and potentially reduce cancer burden through preventive microbiome modulation.

## Author Contributions

J.W. and T.N. designed the work and revised the manuscript. X.Y. prepared the original draft. S.Z. revised the manuscript. All authors contributed to the article and approved the submission.

## Ethic Statement

The authors have nothing to report.

## Conflicts of Interest

The authors declare no conflicts of interest.

## Data Availability

All data are freely available from the corresponding author upon request.
